# Depletion of Histone Demethylase Jarid1A Resulting in Histone Hyperacetylation and Radiation Sensitivity Does Not Affect DNA Double-Strand Break Repair

**DOI:** 10.1371/journal.pone.0156599

**Published:** 2016-06-02

**Authors:** Corina Penterling, Guido A. Drexler, Claudia Böhland, Ramona Stamp, Christina Wilke, Herbert Braselmann, Randolph B. Caldwell, Judith Reindl, Stefanie Girst, Christoph Greubel, Christian Siebenwirth, Wael Y. Mansour, Kerstin Borgmann, Günther Dollinger, Kristian Unger, Anna A. Friedl

**Affiliations:** 1 Department of Radiation Oncology, Ludwig-Maximilians-University of Munich, Munich, Germany; 2 Research Unit Radiation Cytogenetics, Helmholtz Center Munich, Neuherberg, Germany; 3 Institut für Angewandte Physik und Messtechnik, Universität der Bundeswehr München, Neubiberg, Germany; 4 Department of Radiation Oncology, Technical University of Munich, Munich, Germany; 5 Laboratory of Radiobiology & Experimental Radiooncology, University Medical Center Hamburg-Eppendorf, Hamburg, Germany; 6 Tumor Biology Department, National Cancer Institute, Cairo University, Cairo, Egypt; 7 Clinical Cooperation Group ‘Personalized Radiotherapy of Head and Neck Cancer’, Helmholtz Center Munich, Neuherberg, Germany; New York University School of Medicine, UNITED STATES

## Abstract

Histone demethylases have recently gained interest as potential targets in cancer treatment and several histone demethylases have been implicated in the DNA damage response. We investigated the effects of siRNA-mediated depletion of histone demethylase Jarid1A (KDM5A, RBP2), which demethylates transcription activating tri- and dimethylated lysine 4 at histone H3 (H3K4me3/me2), on growth characteristics and cellular response to radiation in several cancer cell lines. In unirradiated cells Jarid1A depletion lead to histone hyperacetylation while not affecting cell growth. In irradiated cells, depletion of Jarid1A significantly increased cellular radiosensitivity. Unexpectedly, the hyperacetylation phenotype did not lead to disturbed accumulation of DNA damage response and repair factors 53BP1, BRCA1, or Rad51 at damage sites, nor did it influence resolution of radiation-induced foci or rejoining of reporter constructs. We conclude that the radiation sensitivity observed following depletion of Jarid1A is not caused by a deficiency in repair of DNA double-strand breaks.

## Introduction

Chromatin structure plays a key role in the regulation of transcription, replication and repair. Thus, deregulation of pathways regulating the epigenome and chromatin structure is an important factor in development of disease, including cancer [1,2]. Cancer cells exhibit characteristic alterations in chromatin structure [3] and recent sequencing analyses of cancer genomes have revealed frequent mutations in genes coding for regulators of the epigenome involved in DNA modifications, histone modifications and chromatin remodeling [2,4]. Among histone modifications, histone lysine methylation gives rise to a complex repertoire of methylation patterns, due to the number of lysine residues that can be methylated and the varying degrees of methylation (me1, me2 and me3). Depending on the site of methylation, its effect on transcription can be activating or repressing. Alterations in genes controlling histone lysine methylation, including histone demethylases, are frequently seen in cancer and have gained attention as potential targets in cancer treatment [5–7]. In addition, the Jumonji (JmjC) domain-containing family of histone demethylases has attracted interest because its members rely on α-ketoglutarate as a co-factor in demethylation and can thus be inhibited by the oncometabolite 2-hydoxyglutarate, a product of mutated isocitrate dehydrogenases IDH1 or IDH2 [8–12] that is frequently observed in a variety of cancer types [13]. Interestingly, in Glioblastoma mutations in *IDH1/2* genes are favorable prognostic factors and there are indications for enhanced radiosensitivity of tumors bearing this mutation [14,15]. This may at least in part be caused by inactivation of the activity of JmjC family histone demethylases, several of which have recently been implicated in genome stability and DNA repair pathways [16–22].

In recent work we observed a loss of di- and trimethylation of histone H3 at lysine 4 (H3K4) and a concomitant loss of active RNA polymerase II in γH2AX-decorated chromatin regions surrounding DNA double-strand breaks (DSB) after treatment with ionizing radiation [23], suggesting that inhibition of transcription in the vicinity of break sites is associated with a loss of activating histone marks. While we were not able to identify the H3K4me3/2 demethylase responsible for this hypomethylation, we observed that the JmjC family histone demethylase Jarid1A (KDM5A/RBP2) accumulates at laser-induced DNA damage sites, making this a strong candidate [23]. Here we report on the effects of Jarid1A depletion on growth characteristics and the cellular response to radiation-induced DNA damage. In unirradiated cells, depletion of Jarid1A did not affect cell growth but resulted in histone hyperacetylation. After irradiation, we did not find indications for a function of Jarid1A in hypomethylation at γH2AX domains or recruitment of other damage response factors to the damage sites, nor did we find indications for a role determining DSB repair efficiency or pathway choice. This is in contrast to the closely related H3K4me3/me2 demethylase Jarid1B, which has been shown to have these functions [17]. Nevertheless, depletion of Jarid1A significantly enhanced sensitivity of cells to ionizing radiation.

## Materials and Methods

### Cell culture and irradiation

HeLa and MCF-7 cells were purchased from DSMZ-German Collection of Microorganisms and Cell Cultures (Braunschweig, Germany), U2OS cells were a kind gift of P. Grigaravicius, Jena [24]. All cell lines were cultivated in RPMI 1640 medium with 10% FBS at 37°C with 5% CO_2_. Irradiation with different doses of X-rays was performed with an Elekta SLI 18 linear accelerator (dose rate 2 Gy/min). For colony formation assays, cells were seeded in 6 well plates 5 h prior to irradiation; for immunofluorescence detection, cells were seeded on glass coverslips the day before irradiation. For irradiation with accelerated 55 MeV carbon ions at the ion microirradiation facility SNAKE [25] at the 14 MV Munich tandem accelerator, cells were seeded 24 h prior to irradiation on 6 μm Mylar foil (pre-coated with Cell-TAK, BD Bioscience) in stainless steel chambers designed as described before [26,27] or, for small angle irradiation, in steel rings [28]. For analysis of correlation between γH2AX and H3K4me3 or RNA Pol II after ion irradiation, cells were irradiated with single ions applied in a linear pattern with 1 μm lateral distance and 5 μm distance between the “lines” thus formed [29], or in a matrix pattern of 5 μm x 5 μm distance [30,31]. In this set-up, ions arrive at a perpendicular angle to the cell layer, which results in a dose of approximately 0.46 Gy per ion hit. For analysis of BRCA1, 53BP1 and Rad51 foci formation, we performed small angle irradiation [28], where the ion beam hits the cell layer at an angle of 10°, allowing visualization of protein accumulations along the track of ion-induced damage. After irradiation cells were incubated in fresh medium for different periods of time.

### RNA interference

All siRNA transfections were performed using Lipofectamine 2000 (Life Technologies) according to the manufacturer’s instructions. All stealth siRNAs were purchased from Life Technologies. For transfection, cells were seeded in 6 well plates and Lipofectamine/ siRNA was added the following day. Per sample, 2.5 μl Lipofectamine and 6.25 μl of the respective siRNA was used, resulting in a siRNA concentration of 50 pmol per well. The stealth scrambled (scr) RNAi was used as control to exclude transfection effects. For depletion of Jarid1A we tested stealth RNAi Jarid1A A1, A2 and A3. If not indicated otherwise, we used a 1:1 combination of the two siRNAs A1 and A3 as this resulted in the highest knock-down efficiencies (A1: CCA AAC UCC AGA UGU UGA UAG AUA U, A3: GAG CCU GAG GUU CUC AGC ACU GAU A). Depending on the purposes of the experiment, cells were irradiated or harvested 72 h later. Efficiency of depletion was verified for every single experiment by Western Blotting and Jarid1A signal was normalized to the sample transfected with scr siRNA.

### Extraction of proteins and Western blotting

Cells were trypsinized, counted and collected by centrifugation (5 min, 500 x g, 4°C). The proteins were extracted with RIPA-buffer (150 mM NaCl, 1% NP-40, 10 mM MDOC, 0.1% SDS, 50 mM Tris pH 8.0) supplemented with PhosSTOP Phosphatase Inhibitor, Complete Mini Protease Inhibitor (Roche) and 5 mM sodium butyrate as HDAC inhibitor on ice. To ensure similar protein amount in the samples, 20 μl buffer per 100 000 cells was used for lysis. The proteins were separated with Laemmli loading dye on 3–8% Tris-Acetate or 12% Bis-Tris NuPAGE gels (Invitrogen). After immunoblotting, membranes were cut and blocked with Roti-Block (Roth), 5% milk or 5% BSA, depending on primary antibody used. Membranes were incubated with primary antibodies in the indicated blocking solution for 1 h before detection with the appropriate goat polyclonal secondary antibodies for 45 min (anti-mouse-HRP and anti-rabbit-HRP; Santa Cruz, sc2004 and sc2005; 0.25 μl/20 ml). Primary antibodies used were mouse monoclonal anti-Jarid1A (Abcam, ab78322; 1:1000), mouse monoclonal anti-Jarid1B (Sigma, SAB1404865; 1:4000), rabbit polyclonal anti-H3K4me3 (Abcam, ab8580; 1:1000), rabbit polyclonal anti-H4K16ac (Millipore, 07–329; 1:1000), rabbit polyclonal anti-H3K9ac (Millipore, 06–942; 1:1000), rabbit polyclonal anti-H3K56ac (Millipore, 07–677; 1:1000), mouse monoclonal anti-H4 (Abcam, ab31830; 1:1000), mouse monoclonal anti-H3 (Millipore, 05–1341; 1:5000), rabbit monoclonal anti-p21 (Cell Signaling, 2947P; 1:1000). Tubulin α detected by the primary antibody mouse monoclonal anti-Tubulin α (Abcam, 7291; 1:6000) served as loading control. Blots were developed with Lumigen ECL Ultra (TMA-6). Chemiluminescence was detected and images were acquired with a CHEMISMART documentation system (Peqlab, Vilber Lourmat) and the Chemi-Capt 5000 software. Quantitative analysis was performed with the Bio-1D software (Vilber Lourmat). The signals were normalized with respect to the scr siRNA transfected, unirradiated samples.

### Immunofluorescence and microscopy

For the immunofluorescence staining experiments cells were grown on glass coverslips or Mylar foils. After treatment cells were fixed for 15 min with 2% paraformaldehyde. Following fixation, cells were washed with PBS, permeabilized with PBS + 0.15% Triton X-100 and blocked with PBS containing 1% BSA and 0.15% Glycine. Cells were then stained with the appropriate primary and secondary antibodies. Primary antibodies used for immunofluorescence were rabbit polyclonal anti-H3K4me3 (Abcam, ab8580; 1:500), rabbit monoclonal anti-Jarid1A/RBP2 (Cell Signaling, 3876; 1:200), mouse monoclonal anti-Jarid1A (Abcam, ab78322; 1:500), rat anti-RNA polymerase II phosphorylated at Ser2 (IgG1) (kindly provided by D. Eick; 1:10), mouse monoclonal anti-γH2AX (Millipore, 05–636; 1:500), rabbit monoclonal anti-γH2AX (Abcam, ab81299; 1:200), rabbit polyclonal anti-53BP1 (Novus, NB100-305; 1:500), mouse monoclonal anti-BRCA1 (Abcam, ab16780; 1:200), rabbit polyclonal anti-Rad51 (Calbiochem, PC130; 1:200). Fluorescence coupled secondary antibodies were goat polyclonal anti-rat Alexa488 IgG (H+L) (Molecular Probes, A11006; 1:500), goat polyclonal anti-rabbit Alexa488 IgG (Molecular Probes, A11034; 1:200) and sheep polyclonal anti-mouse Cy3 F(ab)2 (Dianova, 515-165-062; 1:500). All antibodies were diluted in PBS containing 1% BSA and 0.15% Glycine. Finally, DNA was counterstained with DAPI and cells were mounted in Vectashield for microscopy. Rabbit anti-H3K4me3 (Abcam, ab8580) was tested for specificity with competing peptides in an immunofluorescence and Western Blot assay as described by [32]. Used peptides were H3 unmodified (Abcam, 2903), H3K4me1 (Abcam, 1340), H3K4me2 (Abcam, 7768) and H3K4me3 (Abcam, 1342).

Image acquisition was performed with an inverse epifluorescence microscope (Zeiss AxioObserver Z1,Germany) using a Zeiss LCI Plan Neofluor 63×/1.3 glycerine objective, the software AxioVision 4.8 and a AxioCam Mrm camera (Zeiss). Z-stacks were collected sequentially with 250 nm distance between optical sections.

### Image processing

All z-stack images were deconvolved with Huygens deconvolution software (Scientific Volume Imaging) [33]. Images were further processed using the software ImageJ 1.37c (www.uhnresearch.ca/wcif). To quantify the fluorescence signal of H3K4me3 and Jarid1A in HeLa cells after siRNA transfection, the sum of the intensity values of the pixels in the nucleus (= integrated density) was measured with ImageJ and corrected for background intensity. Mean corrected fluorescence signals of 20 randomly chosen nuclei per sample were determined and normalized with respect to the scr siRNA-transfected sample. Analysis of anti-correlation of γH2AX and H3K4me3 or Ser2-phosphorylated RNA Pol II was performed according to the instructions for the ImageJ-based ICA (intensity correlation analysis) module as described before [23].

### Quantitative analysis of γH2AX, BRCA1, 53BP1 and Rad51 foci

For foci analysis with ImageJ, deconvolved z-stacks were converted to an 8-bit format. The settings of the PlugIn FociPicker 3D [28] had to be optimized for the different antibody stainings by changing the parameters “Tolerance Setting” and “Minimal pixels number in the focus”. The outputs are a 3D FociMask that displays all counted foci in different colours, a result table, where the number of foci per cell and the size of each focus is listed, and a log file as summary of each processed image with file name, parameter setting and number of foci detected. For determination of residual γH2AX, 53BP1 or Rad51 foci, the foci number and size of at least 20 cells per sample were evaluated. For determination of foci forming capability of BRCA1, 53BP1 and Rad51 at least 50 cells with clearly identifiable γH2AX tracks were evaluated. Cells were categorized into (1) full overlap of protein in question with γH2AX track, (2) some foci of protein in question coinciding with γH2AX track, or (3) no foci of protein in question coinciding with γH2AX track.

### MNase digestion

After harvesting the cells (1x10^6^ cells per sample/treatment), nuclei were isolated using a hypotonic buffer (10 mM Hepes pH 7.9, 1.5 mM MgCl_2_, 10 mM KCl, 0.5 mM DTT, 0.1% NP-40). The isolated nuclei were digested at 37°C for different periods of time with 0.5 U MNase (Thermo Scientific) in 650 μl of MNase digestion buffer (50 mM Tris–HCl pH 8.0, 5 mM CaCl_2_). Aliquots of 100 μl were taken every 8 min and MNase activity was inactivated by adding 0.5 M EDTA pH 8.0. DNA isolation was performed by adding SDS to a final concentration of 1% and incubating with RNase A (200 μg/ml) at 37°C for 30 min. Next, Proteinase K (400 μg/ml) was added and the sample was incubated at 55°C for 2 h. Genomic DNA was purified by phenol-chloroform extraction followed by ethanol/acetate precipitation. Similar amounts of the partially digested DNA were loaded on a 2.0% agarose gel and separated by electrophoresis.

### Cell flow cytometry

For cell cycle analysis, HeLa cells were harvested and washed with PBS. For fixation and staining the pellet was resuspended in DNA staining solution I (10 μg/ml RNase, 0.6 mg/ml NaCl, 1 mg/ml Sodium citrate, 0.07% NP-40, 10 μg/ml propidium iodide (PI) in PBS). After incubation for 30 min at RT, DNA staining solution II (15 μg/ml citric acid, 85 μg/ml sucrose, 10 μg/ml PI in PBS) was added. Samples were stored at 4°C until cell cycle data were collected with FACS BD LSR II (Becton Dickinson) and analysed with the free flow cytometry software FlowPy.

For DSB-repair reporter analysis, HeLa pEJ and HeLa pGC cells were harvested 48 h after transfection of pMCV-I-SceI. The cell pellet was washed twice with PBS before fixation with a solution consisting of 3% PFA and 2% glucose in PBS for 10 min on ice. After fixation, PBS was added and cells were washed one more time with PBS. Samples were stored at 4°C in PBS with 10% FBS until measurement of GFP-positive cells with FACS BD LSR II.

### Colony formation assay

To determine the sensitivity to radiation after Jarid1A depletion in a colony formation assay, cells were plated in triplicates at several low densities, depending on the dose. Plates were then irradiated with 0 Gy, 2 Gy, 5 Gy or 10 Gy X-rays and incubated for 10 days. Fixation and staining was done with a solution consisting of 0.3% methylene blue and 80% ethanol. Colonies comprising more than 50 cells were counted. Cell survival curves were calculated with the linear-quadratic model *S0e*^*-ad-bd^2*^, where S0 represents the plating efficiency, *d* the radiation dose, *a*[Gy^-1^] the linear coefficient and *b*[Gy^-2^] the coefficient of the quadratic component. Curve coefficients were calculated with the R-package CFAssay [34,35], using the maximum likelihood method. Curves were compared with the F-test [36].

### DSB repair assay with reporter plasmids

To study the usage of different DSB repair pathways after depletion of Jarid1A, we used a plasmid-based assay. HeLa cells were stably transfected with repair substrates pEJ and pGC [37] to monitor non-homologous end joining (NHEJ) and homologous recombination (HR), respectively. Successful repair after transfection of pMCV-I-SceI, which encodes an endonuclease specific for a sequence in the plasmids, results in expression of green fluorescent protein (GFP). Stably transfected HeLa pEJ and HeLa pGC cells were transfected with scr or Jarid1A A1+A3 siRNA. 24 h later, cells were transfected with the I-SceI expression vector to induce DSB. A vector expressing GFP (pMCC-gfp-P) was used to monitor transfection efficiency in parallel samples. 48 h after transfection of pMCV-I-SceI cells were harvested for flow cytometry analysis.

### Statistical analysis

To examine the differences between the cells transfected with scrambled siRNA and Jarid1A A1+A3 siRNA regarding histone acetylations or DSB repair, an unpaired, 2-tailed t-test was performed using Microsoft Excel 2010. P-values < 0.05 were defined as statistically significant differences.

## Results

### Knock-down of Jarid1A does not affect cell growth

**Fig 1:**
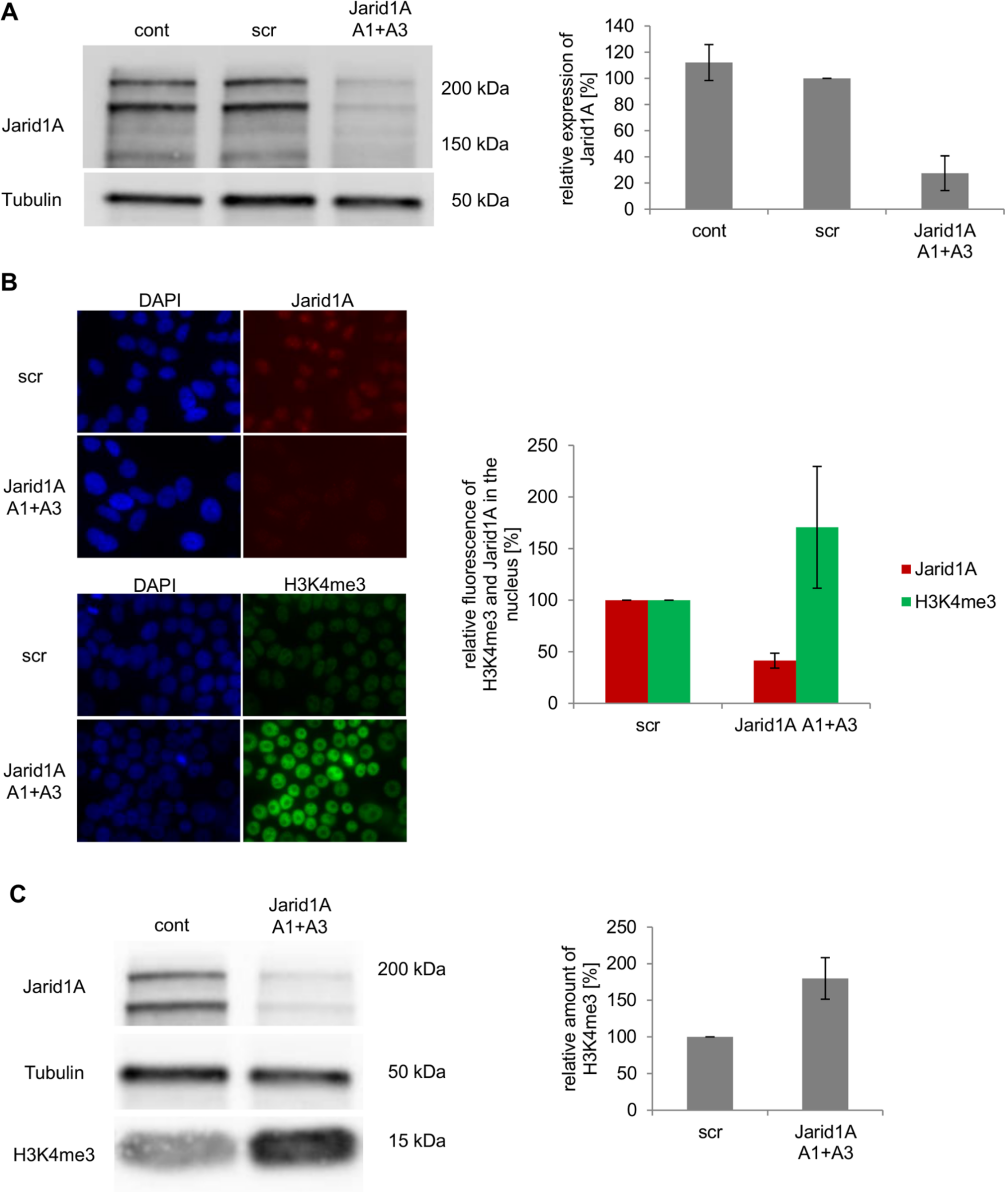
Efficient down-regulation of Jarid1A is associated with global increase of H3K4me3. (A) Decreased levels of Jarid1A in whole cell protein extracts of HeLa cells after siRNA transfection. Western blot images show levels of Jarid1A in untransfected cells (cont), in cells transfected with scrambled siRNA (scr) and in cells transfected with Jarid1A A1+A3 siRNA. Normalized average amount (+/- SD) of Jarid1A protein after siRNA transfection of HeLa cells was determined by quantitative analysis of Western blots of protein extracts obtained in 10 independent experiments. (B) Decreased Jarid1A and increased H3K4me3 signal intensity after indirect immunofluorescence in HeLa cells transfected with Jarid1A A1+A3 siRNA. Microscopic images were obtained at comparable exposure times and display the cell nuclei stained with DAPI in blue, the protein Jarid1A in red and the histone modification H3K4me3 in green. The graph indicates normalized average fluorescence of Jarid1A and H3K4me3 in nuclei of 20 randomly chosen cells. (C) Increase of H3K4me3 in whole cell protein extracts of HeLa cells after depletion of Jarid1A. Western blot images show levels of Jarid1A and H3K4me3. Average levels of H3K4me3 (+/- SD) were determined by quantitative analysis of protein extracts obtained in 3 independent experiments via Western Blot.

Alternative splicing yields two isoforms of Jarid1A [38] the functional difference of which is poorly described. Both isoforms (expected sizes 192 kD and 186 kD) are detected in HeLa cells (Fig 1A), as well as in MCF-7 and U2OS cells (panel A in S1 Fig) and total expression of Jarid1A protein is comparable in these cell lines (panel A in S1 Fig). Using stealth siRNA, efficient knock-down of both isoforms is achieved in HeLa cells. At 72 h after transfection, protein levels in whole cell extracts are reduced to 27.4 ± 13.3% (Fig 1A). Similar depletion of Jarid1A was obtained in U2OS and MCF-7 cells (panel A in S1 Fig). Jarid1A knock-down also reduces the intensity of an uncharacterized band of about 130 kD, which presumably represents a degradation product of Jarid1A. If not stated otherwise, for our experiments we transfected cells with a 1:1 combination of Jarid1A siRNAs A1 and A3, but similar reductions were seen after using each of these siRNAs alone (panel B in S1 Fig). Drastic reduction of nuclear Jarid1A after transfection with Jarid1A siRNA is also evident from immunofluorescence (IF) detection (Fig 1B). As a result of Jarid1A knock-down, cellular H3K4me3 levels increase about 1.7-fold, as seen by immunofluorescence (Fig 1B) and Western Blotting (Fig 1C). Comparable increases were seen in U2OS and MCF-7 cells (panel C in S1 Fig). The observed increase of H3K4me3 cannot be explained by differences in H3 expression (panel D in S1 Fig). The antibody used here is very specific for H3K4me3 in IF, while in Western Blotting also H3K4me2 is detected (panel E in S1 Fig).

**Fig 2:**
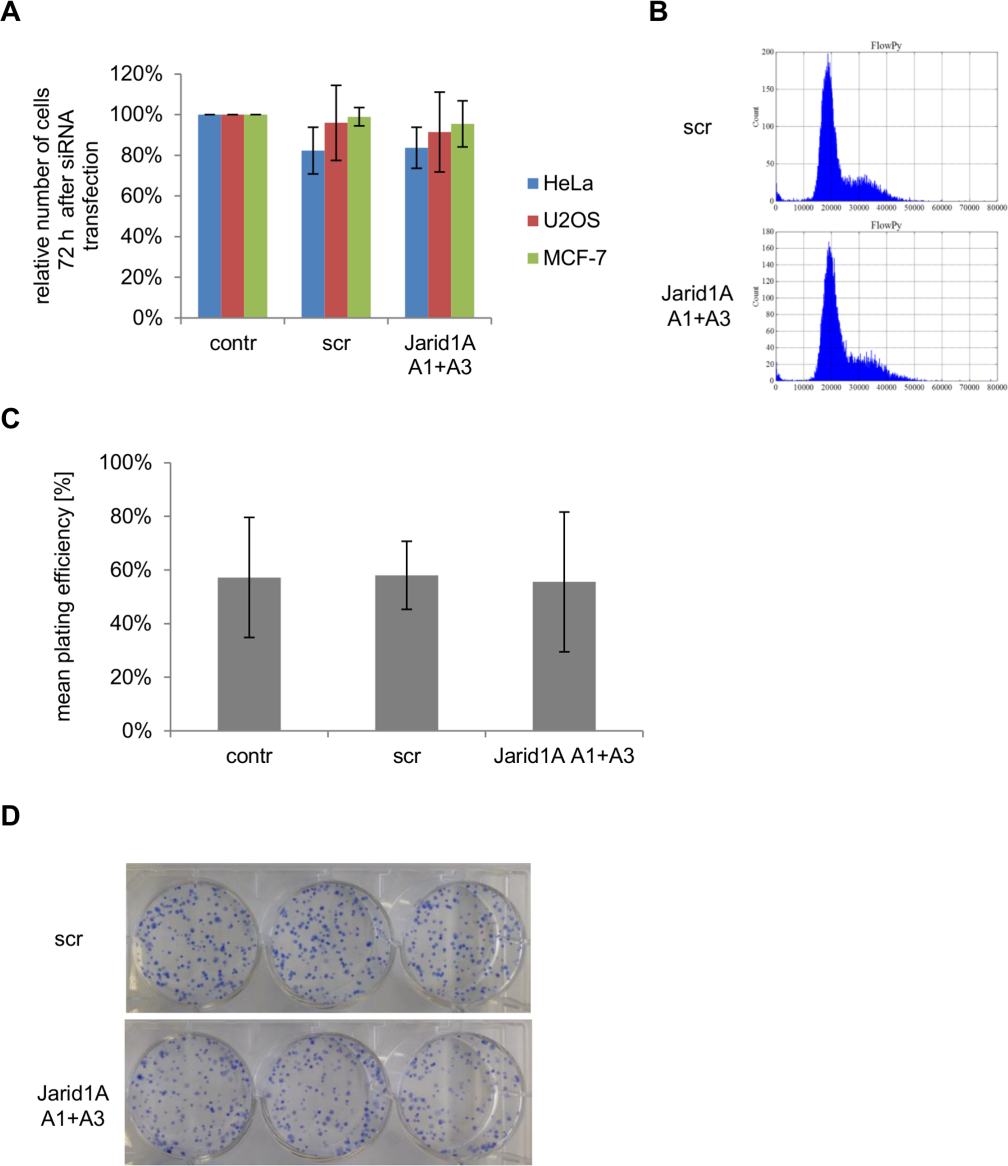
Jarid1A depletion does not affect cell proliferation. (A) No difference in the cell number 72 h after transfection with scr or Jarid1A siRNA. In all experiments involving protein extracts, the cell number of the different transfection samples was determined prior to protein extraction. Indicated are mean cell numbers (+/- SD) of HeLa cells (12 experiments), U2OS cells (3 experiments) and MCF-7 cells (2 experiments), each normalized with respect to the untransfected control samples. (B) Consistent cell cycle distribution after down-regulation of Jarid1A. 72 h after siRNA transfection HeLa cells were harvested, stained with PI and analyzed by flow cytometry. (C) Constant plating efficiency of the transfected cells. After incubating transfected and control HeLa cells for 10 days, colonies were stained with methylene blue and the number of colonies was determined. Graph indicates the mean plating efficiency (+/- SD) of 3 independent colony formation assays. (D) Methylene blue stained colonies 10 days after seeding of 300 cells per well.

It has been reported that Jarid1B and Jarid1A can compensate for each other [39,40]. We therefore investigated Jarid1B protein levels after Jarid1A knock-down and did not detect a concomitant increase (panel F in S1 Fig). Although the accuracy of expression levels obtained for different genes in microarray analyses for the purpose of a gene-to-gene comparison is limited due to small differences in GC content of array probes and subsequent variation in hybridization affinities, our data suggest a markedly higher expression of Jarid1B mRNA in untreated HeLa cells compared to the three other Jarid1 family members (normalized linear expression values: Jarid1A: 78.4, Jarid1B: 535.1, Jarid1C: 133.2 and Jarid1D: 86.6). In order to estimate if the ratio between Jarid1A and Jarid1B expression also differs in the cell lines tested here at the protein level, we performed quantitative Western blot analysis of Jarid1B (panel F in S1 Fig) and observed comparable values for the ratio of Jarid1A/Jarid1B band intensities (S1 Table). Unfortunately, we could not study the effects of Jarid1B knock-down since in our hands cells detached rapidly once Jarid1B levels were strongly reduced (data not shown). In contrast, cell yield at 72 h after transfection was the same with scr siRNA or Jarid1A siRNA (Fig 2A), demonstrating that in Hela, MCF-7 or U2OS cells strong reduction of cellular Jarid1A levels does not affect short-term viability and proliferation. This is in contrast to published data reporting on induction of senescence, accumulation in G1 cell cycle phase and/or induction of cyclin-dependent kinase inhibitors, such as p21, upon depletion of Jarid1A [41–45]. In line with our viability data we did not find indications for substantial up-regulation of p21 in our cell lines (S2 Fig). In addition, cell cycle distribution did not differ between HeLa cells transfected with scr siRNA and Jarid1A siRNA at 72 h after transfection (Fig 2B). Long-term viability and growth after Jarid1A knock-down was investigated in HeLa cells by determining the plating efficiency in colony formation experiments. Both plating efficiency (Fig 2C) and average colony size (Fig 2D) were unaffected by Jarid1A knock-down. In conclusion, under conditions leading to strong reduction of Jarid1A level and concomitant increase in cellular H3K4me3 levels, cellular viability and growth characteristics remain unaffected in the cell lines tested here.

### Depletion of Jarid1A results in histone hyperacetylation

**Fig 3:**
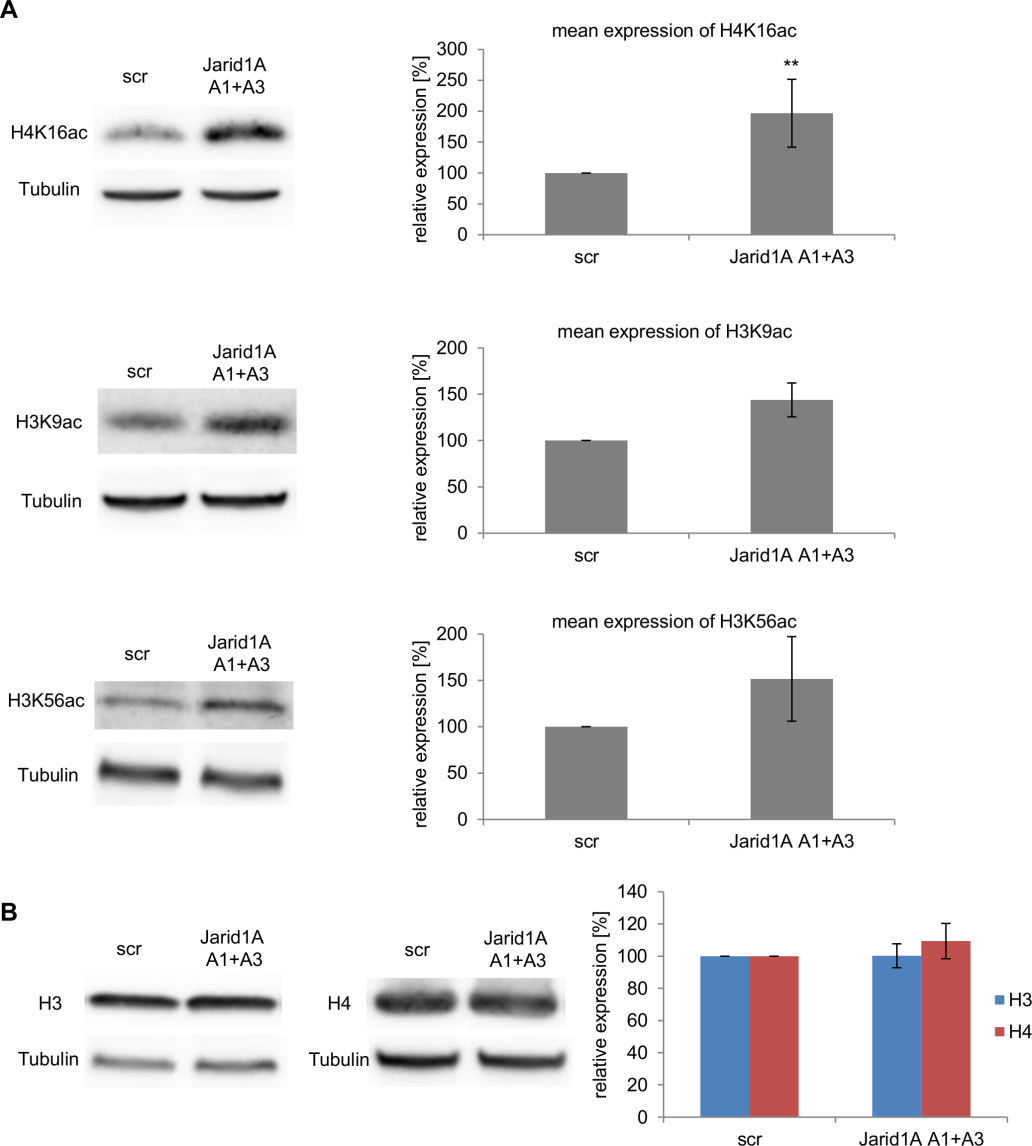
Depletion of Jarid1A results in histone hyperacetylation. (A) Increase of H4K16ac, H3K9ac, and H3K56ac in whole cell protein extracts of HeLa cells after siRNA transfection. Western blot images show levels of the histone modifications in cells transfected with scrambled siRNA (scr) and Jarid1A A1+A3 siRNA. Graphs indicate the means (+/- SD) from 5 independent experiments for H4K16ac and 3 independent experiments for H3K9ac and H3K56ac after quantitative analysis of Western blots. The effect of H4K16ac is statistically significant (p = 0.0035). (B) The amounts of the histones H3 and H4 are not affected by down-regulation of Jarid1A. Indicated are means (+/- SD) from 3 experiments.

Jarid1A interacts physically with several histone deacetylase complexes [46–48]. In addition, pathway enrichment analysis of preliminary microarray experiments suggested deregulation of histone deacetylation pathways upon Jarid1A depletion (data not shown). We therefore investigated the influence of Jarid1A knock-down on the levels of acetylation at H3K9, H3K56 and H4K16. As depicted in Fig 3A, acetylation levels of H4K16 increased significantly by about 100% at 72 h after siRNA transfection (p = 0.0035). Increased acetylation was also seen for H3K9 and H3K56, albeit statistical significance was not reached. These increases were not due to an increase in the amount of histones H3 and H4 after Jarid1A depletion (Fig 3B). In spite of the histone hyperacetylation, we did not observe increased susceptibility to micrococcal nuclease in chromatin isolated from Jarid1A-depleted cells (S3 Fig), in accordance with earlier reports, e.g. [49,50].

### Depletion of Jarid1A sensitizes cells to ionizing radiation

**Fig 4:**
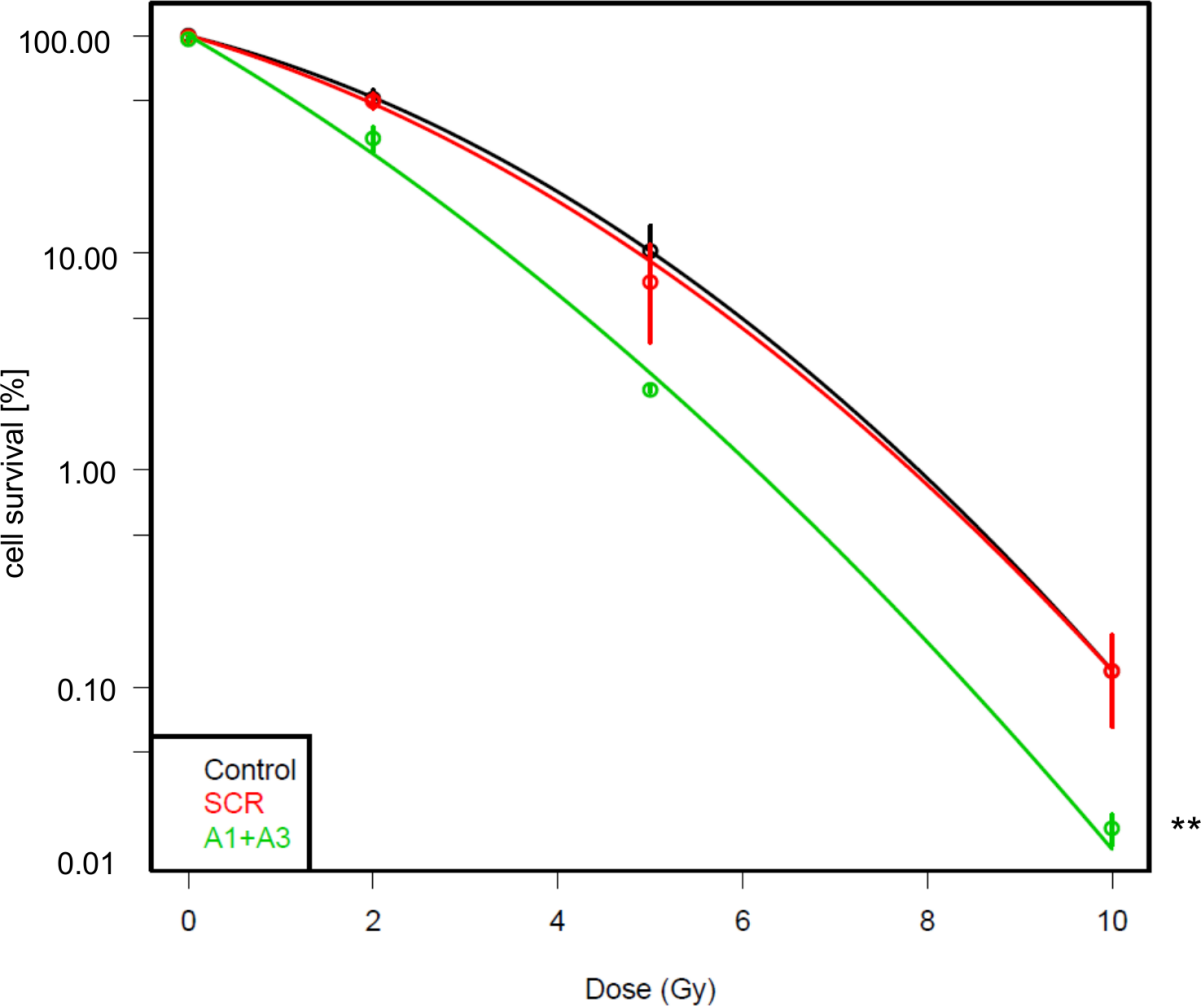
Depletion of Jarid1A enhances radiosensitivity. Survival fraction of the differently treated HeLa cells irradiated at 72 h after siRNA transfection. Cells were irradiated with 0 Gy, 2 Gy 5 Gy or 10 Gy X-rays and incubated for 10 days before fixation and methylene blue staining of colonies. For every dose the mean value of the cell survival (+/- SD) of 3 independent colony forming assays is shown. Data were fitted with a linear-quadratic model and statistical significance was determined by F test. After Jarid1A depletion, survival is significantly reduced as compared to scr siRNA transfected cells (p = 0.0025) and untransfected controls (p < 0.0001).

It is well established that treatment of cells with inhibitors of histone deacetylases (HDACi) or depletion of HDACs enhances radiosensitivity [51,52]. While a variety of cellular pathways appear to be affected by treatment with HDACi, radiosensitization is also observed at low concentrations of HDACi where the only discernible effect is chromatin hyperacetylation, suggesting a direct influence of hyperacetylation on radiation sensitivity [53]. Therefore, we set out to investigate the effect of Jarid1A depletion on survival of cells after X-irradiation in a colony formation experiment. We observed a moderate, but highly significant sensitization of cells after Jarid1A depletion with siRNAs A1+A3 as compared to untransfected (p < 0.0001) and control-transfected (p = 0.0025) cells (Fig 4). Sensitization was also observed after transfection with each of these siRNAs alone, thus ruling out off-target effects (S4 Fig). Hence, Jarid1A activity does play a role in the cellular resistance to radiation damage.

### Jarid1A is not essential for radiation-induced loss of H3K4me3 at γH2AX-decorated chromatin domains nor for recruitment of repair proteins

In previous work we observed a loss of H3K4me2/3 and of elongation-active RNA Pol II in ionizing radiation-induced foci (IRIF) of γH2AX [23]. To investigate if Jarid1A is involved in this process, we treated HeLa cells 72 h after transfection with scr or Jarid1A siRNA with carbon ions at the ion microirradiation facility SNAKE [25]. After irradiation with single carbon ions, γH2AX IRIF forming along the ion tracks were visualized by immunofluorescence and 3D microscopy. At 1 h after irradiation, underrepresentation of H3K4me3 at γH2AX foci is comparable in both samples (panel A in S5 Fig). Similarly, underrepresentation of elongating Ser2-phosphorylated RNA polymerase II is not affected by depletion of Jarid1A (panel B in S5 Fig). We conclude that depletion of Jarid1A does not disturb H3K4me3 demethylation at damage sites.

**Fig 5:**
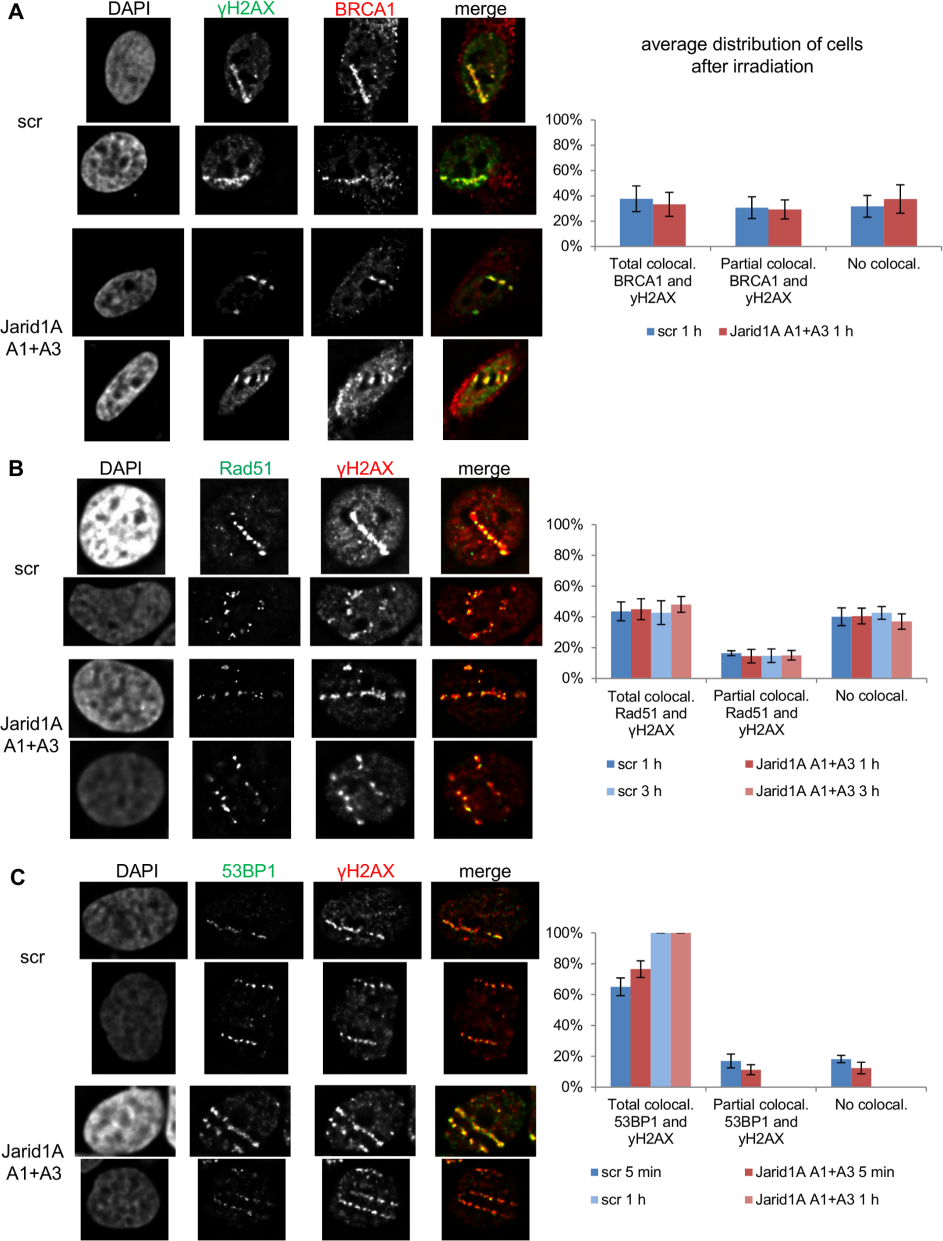
Formation of radiation-induced protein foci is not affected by depletion of Jarid1A. Detection of repair foci in scr or Jarid1A A1+A3 siRNA transfected HeLa cells after ion microirradiation. 72 h after transfection cells were irradiated in a small angle (10°) with 55 MeV carbon ions. After incubation, cells were fixed and indirect immunofluorescence was performed to detect the foci formation of γH2AX and BRCA1 (A), Rad51 and γH2AX (B), or 53BP1 and γH2AX (C). For each sample, two typical cells are depicted. For quantitative evaluation, cells with γH2AX tracks were divided in 3 groups, depending on whether the protein in question formed foci that overlapped with γH2AX foci along the whole track, or occasional foci that overlapped with some of the γH2AX foci in the track, or no overlapping foci. Indicated are means (+/- SEM) from at least 50 evaluated cells.

It has been suggested that histone hyperacetylation affects repair of radiation-induced DSB [51,53]. In particular, a crucial role for H4K16ac in DSB repair pathway choice was uncovered: H4K16 acetylation antagonizes 53BP1 binding to chromatin and 53BP1 foci formation [54,55], thereby enabling BRCA1 accumulation, end resection and thus initiation of repair via homologous recombination. Others have reported that CHD4, with which Jarid1A was found to interact [47], is required for accumulation of BRCA1 at damage sites [56]. We therefore investigated BRCA1, 53BP1 and Rad51 IRIF formation after irradiation of HeLa cells with 55 MeV carbon ions in a small-angle irradiation geometry that allows visualizing protein accumulation and IRIF formation along the ion-induced damage track [28,57]. Importantly, depletion of Jarid1A (and concomitant hyperacetylation of H4K16) has no effect on frequency or size of BRCA1 foci at 1 h after ion irradiation (Fig 5A). Both, in samples transfected with scr siRNA and samples transfected with Jarid1A siRNA, of all cells with one or more γH2AX tracks, about 1/3 show colocalization of γH2AX and BRCA1 along the entire track, about 1/3 show occasional BRCA1 foci along the γH2AX track, and about 1/3 do not show BRCA1 foci. The presence of BRCA1 at the damage sites suggests initiation of end resection and homologous recombination [58]. Indeed, accumulation of recombination factor Rad51 along the γH2AX tracks is comparable in scr siRNA and Jarid1A siRNA transfected cells at 1 h and 3 h after ion irradiation (Fig 5B). Also 53BP1 accumulation at γH2AX tracks was not affected by Jarid1A depletion (Fig 5C). At 1 h after ion irradiation, 100% of cells with γH2AX tracks exhibited colocalization with 53BP1 along the entire track, independent of Jarid1A status. We also investigated earlier time points (5 min) where due to ongoing recruitment of 53BP1 full colocalization is not yet obtained and did not find differences between src siRNA and Jarid1A siRNA transfected samples. We conclude that depletion of Jarid1A and concomitant hyperacetylation of H4K16 do not affect recruitment of 53BP1 and BRCA1 at ion irradiation-induced damage sites. Furthermore, down-stream recombinational repair steps appear not to be affected.

We did also not find differences in the early steps of DSB detection and signaling, since γH2AX foci formed equally fast in Jarid1A depleted and control cells after carbon ion or X-irradiation. Foci formation after irradiation with carbon ions was evaluated 5 min (panel A in S6 Fig) and 2 min (panel B in S6 Fig) after irradiation. Because of the experimental set-up, the shortest post-irradiation time possible is 2 min. Also after this short time, no discernible difference in γH2AX formation could be found (panel B in S6 Fig). Similarly, 15 minutes after X-irradiation, γH2AX foci have comparable brightness in both samples (panel C in S6 Fig). We conclude that despite its influence on the cellular H4K16 acetylation levels, Jarid1A is not involved in the pathways regulating inhibition of transcription and accumulation of DSB repair factors at damage sites.

**Fig 6:**
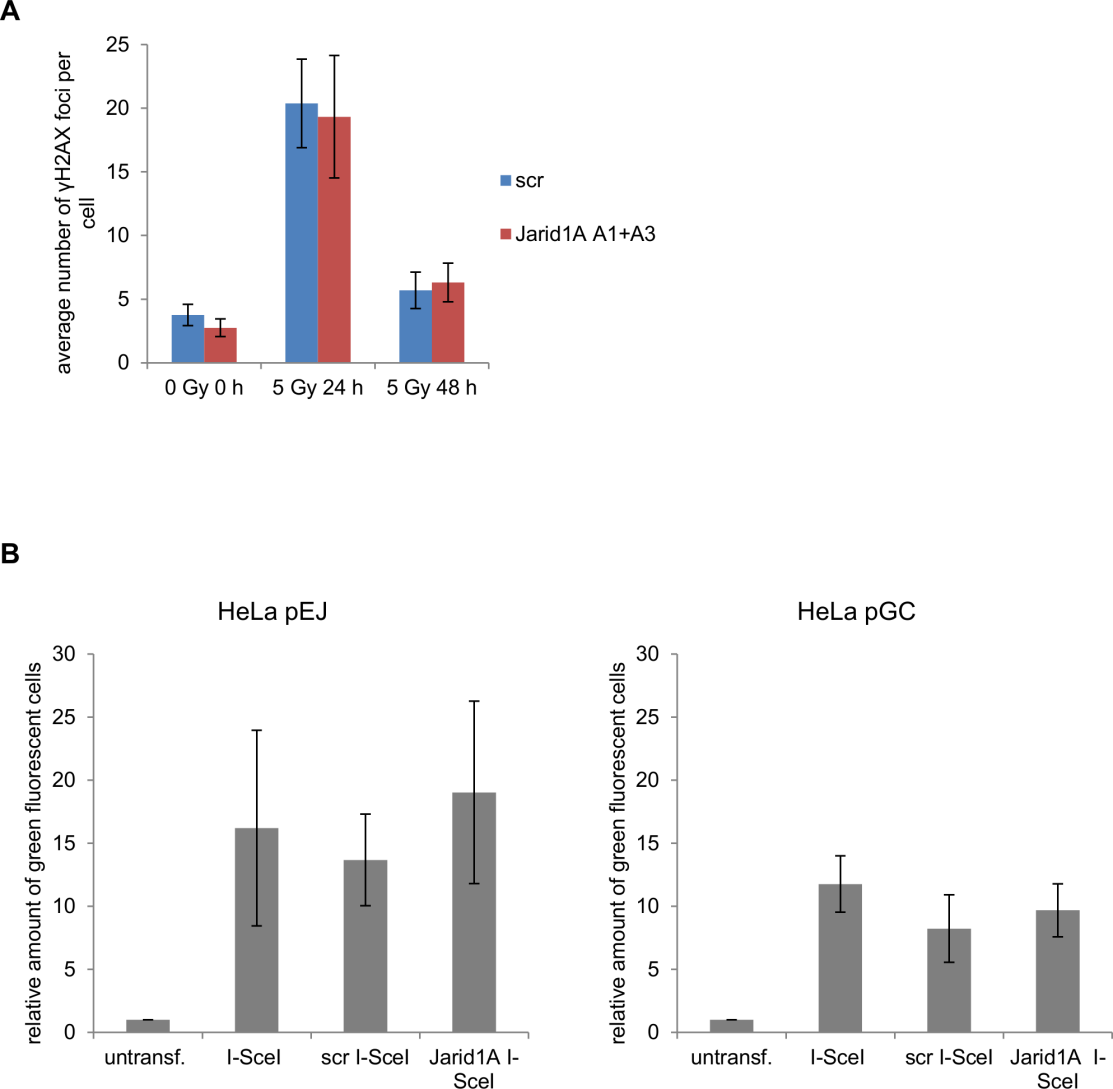
Residual γH2AX foci and reporter plasmid-based DSB repair events are not affected by depletion of Jarid1A. A) Mean background and residual foci number (+/- SEM) in at least 20 cells per sample after 5 Gy X-rays. Cells were fixed before and 24 h or 48 h after irradiation and indirect immunofluorescence was performed. Semi-automatic detection and characterization of spontaneous and residual γH2AX foci in the cells was performed by using the PlugIn FociPicker3D [28]. B) Relative number of GFP expressing cells, normalized to the frequency in untransfected HeLa pEJ (left panel) and pGC (right panel) control cells, after transfection of I-SceI expression plasmid into untreated cells and cells treated with scr siRNA or Jarid1A A1+A3 siRNA. Indicated are means ± SEM of 5 independent experiments.

Determination of residual IRIF after repair incubation is a frequently used method for estimation of DSB repair efficiency [59,60]. To investigate the influence of Jarid1A depletion on DSB repair efficiency, we analyzed the resolution of γH2AX foci after X-irradiation of HeLa cells with 5 Gy (Fig 6A). A comparable frequency of about 3 background γH2AX foci was seen in unirradiated Jarid1A-depleted and scr siRNA controls. At 24 h after X-irradiation the number of γH2AX foci was still considerably enhanced, while at 48 h it was only slightly higher than in unirradiated samples (Fig 6A), suggesting successful completion of DSB repair at most sites. The frequency of residual foci at 24 h and 48 h after irradiation did not differ between Jarid1A-depleted cells and scr siRNA controls (Fig 6A). Similarly, the frequency of 53BP1 and Rad51 background foci or residual foci after X-irradiation was not affected by Jarid1A depletion (S7 Fig).

To verify that DSB repair is not reduced in Jarid1A-depleted cells, we in addition used a plasmid-based DSB repair assay in which successful repair of I-SceI-induced DSB leads to GFP expression that can be detected by flow cytometry [37,61,62]. Reporter plasmids pEJ and pGC were used to monitor NHEJ and HR events, respectively, by stable integration in HeLa cells. After transfection of the I-SceI expression plasmid, the number of GFP-expressing cells increased 16.2-fold in HeLa pEJ and 11.8-fold in HeLa pGC cells (Fig 6B). Comparable frequencies of productive DSB repair events were observed after transfection with scr siRNA and Jarid1A A1+A3 siRNA (Fig 6B), thus substantiating that depletion of Jarid1A under the conditions used in this work does not reduce DSB repair efficiency.

## Discussion

In the present work we investigated the role of histone demethylase Jarid1A in cell proliferation and radiation response by siRNA-mediated depletion of Jarid1A. All experiments reported were performed 72 h after transfection with Jarid1A siRNA under comparable levels of residual amount of protein. Under these conditions global trimethylation of H3K4 increased by about 70%, thus demonstrating efficient functional depletion of Jarid1A demethylation activity. We can, however, not exclude that complete deletion of Jarid1A (as obtained for example by knock-out) would lead to further effects in addition to the effects described here. Hou et al. also reported a drastic increase of global H3K4me3 after depletion of Jarid1A, albeit without showing quantitative data [63]. Others observed similar increases of global H3K4 trimethylation after depletion of Jarid1B [64,65], but not after depletion of Jarid1C [66]. So far, a systematic comparison of the role of the members of the Jarid1protein family for global H3K4me3 levels is lacking.

Jarid1A has been implicated in senescence pathways, both positively and negatively. Several reports describe a loss of clonogenic potential, accumulation in G1 cell cycle phase, increased senescence-associated β-galactosidase activity and/or increased levels of cyclin-dependent kinase inhibitors, such as p21 and p27, after knock-down of Jarid1A in a variety of cell lines [41–45]. Others reported that depletion leads to reduced proliferation only in cell lines with Jarid1A amplification, but not in lines with normal Jarid1A expression [63]. Sharma et al. reported normal proliferation of PC9 lung cancer cells after knock-down of Jarid1A [67]. It has also been noted that Jarid1A knock-out mice exhibit a grossly normal phenotype, thus arguing against a general strong reduction of cell proliferation upon Jarid1A depletion [68,69]. Even a pro-senescent function of Jarid1A was described in some systems [40]. In our hands, depletion of Jarid1A in HeLa, U2OS and MCF-7 cells did not affect cell viability or proliferation. We consider it unlikely that this lack of effect is due to insufficient depletion of Jarid1A, since comparable levels of depletion were described in the publications mentioned above, and since considerable increase of cellular H3K4me3 levels was observed. In HeLa cells p53 levels are low due to enhanced degradation, and concomitantly p21 levels are low. U2OS and MCF-7 cells exhibit wild-type p53 function. Protein level of p21 is attenuated in U2OS cells due to translational inhibition [70,71], but normal in MCF-7 cells. Thus, lack of senescence induction in our cells cannot solely be attributed to deficiencies in the p53-p21 axis.

Cross-regulations of histone modifications are increasingly acknowledged [72]. Specifically, crosstalk between H3K4me2/me1 demethylation by LSD1 (KDM1a) and histone acetylation has been well established. Depletion of LSD1 resulted in down-regulation of HDACs [73] and hyperacetylation at H4K16ac, H3K56ac, and H3K14ac [74]. Potential crosstalk involving demethylases of the Jarid family is less well investigated. Inhibition of histone deacetylases has been reported to result in reduced Jarid1A activity and to phenocopy Jarid1A depletion [67,75–77], but to our knowledge the effect of Jarid1A depletion on histone acetylation has not yet been investigated in detail. We observed a significant increase of acetylation at H4K16 upon depletion of Jarid1A. In addition, our data suggest hyperacetylation at H3K9 and H3K56. These three sites are known targets of HDAC1 and HDAC2 [51,78], with which protein Jarid1A was shown to physically interact [46,79]. In addition, Jarid1A was demonstrated to interact with the HDAC1/2-containing Sin3B and NuRD protein complexes [46–48,68]. It has been proposed that Jarid1A, due to its chromatin-binding PHD finger and ARID domains, may act to recruit Sin3B and NuRD complexes to chromatin [47], hence depletion of Jarid1A may result in reduced HDAC activity at chromatin. Further experiments will be required to elucidate the interplay between Jarid1A and histone deacetylation. At present, we can also not exclude a role for histone acetyl-transferases in the observed hyperacetylation.

Histone hyperacetylation associated with Jarid1A depletion did not discernibly alter nucleosome occupancy, as determined by MNase sensitivity in bulk chromatin. Similarly, others found that histone hyperacetylation does not translate into altered MNase digestion pattern [49,50,80]. We note that MNase digestion of bulk chromatin is a rather insensitive method [81], and an influence on local nucleosome occupancy patterns cannot be excluded. Histone acetylation has been associated with open chromatin states and H4K16ac has been shown in vitro to reduce formation of compact chromatin fibers [82,83], although it may not visibly influence chromatin compaction at all size scales of analyzed structures [84].

Radiation-induced γH2AX foci locate preferentially in euchromatic regions [53,85–87], which has been interpreted in terms of increased DSB formation in regions with less compacted chromatin. It could, however, not be excluded in these studies that the detection of DSB and/or the cellular response reactions differ in a way that results in suppression of foci formation in compacted heterochromatin. By using labeling of DSB ends Takata et al. showed [88] that radiation-induced DSB formation is reduced in artificially condensed chromatin. Pretreatment with HDAC inhibitors has been reported to lead to enhanced γH2AX signals after irradiation [51,89–92], but it is difficult to differentiate effects of enhanced DSB induction, enhanced DSB detection and subsequent signaling, or reduced dephosphorylation of γH2AX and repair of DSB. Others observed enhanced H2AX phosphorylation at Ser139 upon H4K16 hypoacetylation [93], suggesting a complex balance between H4K16ac levels and γH2AX levels. We did not find indications for enhanced γH2AX signals shortly after irradiation of Jarid1A depleted cells and conclude that histone hyperacetylation as is seen after Jarid1A depletion does not fully phenocopy the effects of HDAC inactivation.

We investigated the influence of Jarid1A depletion and H4K16 hyperacetylation on recruitment of DNA damage response and repair factors to IRIFs induced by irradiation with carbon ions. Due to the increased linear energy transfer (LET) of carbon ions as compared to photon irradiation, about 30 DSB are formed per 10 μm track length through the nucleus [25,94], leading to a chain of IRIF formed along the track. Compared to photon irradiation, the increased damage density of carbon ion irradiation facilitates specific detection of IRIF. It should, however, be noted that the damage densities occurring are still physiologically relevant and encountered in the natural and medical environment. This is in contrast to laser microirradiation where local damage densities may be extremely high, allowing the microscopic detection of recruitment to the damage sites even of proteins that accumulate to each DSB site in very low number [95–97].

Acetylation of H4K16 plays a key role in DSB repair pathway decision, since chromatin recruitment of 53BP1, which results in blocked end resection and use of NHEJ repair, is disturbed by the acetyl moiety [54,55]. Thus, one would expect reduced recruitment of 53BP1 to radiation-induced foci upon hyperacetylation, as has been shown by several authors [54,55,92] following HDACi treatment. Others, however, found unaltered 53BP1 recruitment upon hyperacetylation [98] or even impaired 53BP1 recruitment following hypoacetylation of H4 [93,99]. It has been proposed that kinetically different chromatin alterations affect pathway choice, which implies that differing time points of analysis may explain some of the observed discrepancies [98]. In our hands, recruitment of 53BP1 to ion induced damage sites was not affected by Jarid1A depletion. We also did not detect effects on recruitment of pro-recombination factors BRCA1 and Rad51. Published data on BRCA1 recruitment to damage sites under conditions of HDAC inactivation are also quite inconsistent. Some authors observe increased BRCA1 binding to chromatin and foci formation after HDAC inactivation [55], while others report on reduced BRCA1 recruitment after treatment with HDACi [92,98].

Analysis of the number of residual IRIF after repair incubation is a frequently used tool for assessing DSB repair efficiency [59,60]. Residual γH2AX foci at 24 h and 48 h after X-irradiation did not differ between cells scr siRNA and Jarid1A siRNA transfected cells, suggesting that the increased radiation sensitivity of Jarid1A-depleted cells observed in our work cannot be explained by altered DSB repair efficiency. Also the number of residual 53BP1 and Rad51 foci did not differ between the samples, arguing against a difference in pathway use. These results were corroborated by using a plasmid-based repair assay [37,61,62] able to differentiate between end joining and gene conversion repair pathways. The absence of a discernible DSB repair defect is also in contrast to the situation after treatment with HDAC inhibitors or depletion of HDACs, where attenuated DSB repair has been reported [51,53].

Finally, we also investigated whether Jarid1A, which we found previously to accumulate at laser-induced damage sites, has a function in demethylation of H3K4me3/me2 in γH2AX-decorated chromatin regions and loss of active RNA polymerase II from these regions [23]. Our negative results are compatible with observations by Li and coworkers [17] who found that H3K4me3 levels in the vicinity of enzyme-induced DSB are largely regulated by Jarid1B. Although functional overlap of Jarid1A and Jarid1B has been found in certain instances [39,40], the roles of these closely related demethylases in the DNA damage response appears to differ. In line with this assumption, depletion of Jarid1B or loss of its demethylase activity results in reduced accumulation of DSB repair factor BRCA1 at damage sites [50], contrary to the effects of Jarid1A depletion observed in our work.

As noted above, we cannot exclude the possibility of further effects occurring upon complete depletion of Jarid1A. Since under the conditions used here histone hyperacetylation and radiosensitivity increase, while recruitment of DSB signaling and repair factors and DSB repair itself remain unaffected, we propose that the observed hyperacetylation does not affect DSB repair and that the observed radiosensitivity cannot be explained by DSB repair deficiencies. Thus, the mechanistic basis of the significantly increased radiosensitivity seen upon depletion of Jarid1A remains to be elucidated. In addition to the absence of effects on DSB signaling and repair as reported here, we did also not find indications for alterations in cell cycle regulation or induction of apoptosis (data not shown). Given that Jarid1A is involved in transcriptional regulation, we currently investigate the influence of Jarid1A depletion on radiation-induced gene expression alterations.

## Supporting Information

S1 Fig Efficient down-regulation of Jarid1A is associated with a global increase of H3K4me3. (A) Decreased levels of Jarid1A in whole cell protein extracts of HeLa, MCF-7 and U2OS cells after siRNA transfection. Western blot images show levels of Jarid1A in untransfected cells (cont), in cells transfected with scrambled siRNA (scr) and in cells transfected with Jarid1A A1+A3 siRNA. (B) Highest knock-down efficiency is accomplished with a combination of the siRNAs Jarid1A A1 and A3. An unspecific band at about 170 kD seen in certain gel settings is not affected by siRNA transfection. The graph depicts results of quantitative evaluation of the blot shown on the left side. (C) Increase of H3K4me3 in whole cell protein extracts of MCF-7 cells and U2OS cells after knock-down of Jarid1A. Western blot images show levels of Jarid1A and H3K4me3 in cells transfected with scr siRNA and Jarid1A A1+A3 siRNA 72 h after transfection. Numbers give levels of H3K4me3 normalized to scr sample after quantitative analysis. (D) Increase of H3K4me3 after Jarid1A depletion is not due to alterations in level of histone H3. Parallel samples of protein extracts of cells transfected with scr siRNA and Jarid1A A1+A3 siRNA were loaded. The low molecular weight region of the Western Blot was cut into 2 halves to visualize H3 and H3K4me3. Numbers give levels of H3K4me3 normalized to scr sample after quantitative analysis. (E) Specificity of the H3K4me3 antibody was established by peptide competition assays via immunofluorescence and Western Blot. The graph at the left shows the mean x-fold change of exposure time (+/-SD) from 5 randomly chosen positions in the immunofluorescence samples after incubation of the antibody with different peptides. Efficient blocking of the antibody is only seen with H3K4me3 peptides. For the Western Blot, the relative signals of H3K4me3 after incubation of the antibody with the different peptides were calculated and are represented in the right graph. The antibody is efficiently blocked by peptides H3K4me2 and H3K4me3. (F) Left panel: Level of Jarid1B protein is not increased by depletion of Jarid1A. Right panel: Comparable levels of Jarid1B protein in HeLa, U2OS and MCF-7.
(TIF)

S2 Fig Depletion of Jarid1A does not lead to strong induction of p21. Expression of p21 after depletion of Jarid1A in HeLa, MCF-7 and U2OS cells, 72 h after transfection with scr or Jarid1A siRNA. A representative sample and its quantitative evaluation are shown. Cont = untransfected control.
(TIF)

S3 Fig MNase accessibility is not affected by Jarid1A depletion. Analysis of chromatin accessibility by MNase digestion of isolated nuclei. After different incubation periods with 0.5 u MNase, comparable amounts of partially digested DNA were loaded onto an agarose gel. The emerging ladder of mono- and oligonucleosomes is comparable in both samples indicating regular nucleosome distribution in bulk chromatin after Jarid1A depletion.
(TIF)

S4 Fig Depletion of Jarid1A enhances radiosensitivity. Colony formation experiment with Hela cells transfected with JaridA1 siRNAs A1 or A3 or a combination thereof, as well as untransfected controls and cells transfected with scr siRNA. Cells were irradiated 72 h after siRNA transfection with 0 Gy, 2 Gy, 5 Gy or 10 Gy X-rays. Cells were incubated for 10 days before fixation and methylene blue staining of colonies. Data show that the different Jarid1A siRNAs lead to comparable sensitization as compared to controls.
(TIF)

S5 Fig Depletion of Jarid1A does not affect anti-correlation of γH2AX and H3K4me3 or active RNA Pol II, respectively, after ion irradiation. HeLa cell transfected with scr or Jarid1A A1+A3 siRNAs were subject to ion microirradiation with single carbon ions applied in line patterns (lateral distance between single ion hits 1 μm, distance between “lines” 5 μm). Cells were incubated for 1 h before fixation and indirect immunofluorescence detection of γH2AX and H3K4me3 (A) or elongation-proficient RNA Pol II Ser2-p (B). Correlation analysis was done as described [23]. In all panels the top rows show single slices of 3D microscopic images (red channel, green channel and merge). In addition, to determine positive or negative correlation between signal intensities in both channels for each pixel, the product of the mean (PDM) map is shown. In the PDM maps, negative correlation at positions of γH2AX foci is visualized by pink signals; positive correlation is shown by green signals, whereas black indicates random distribution of both signals. In the second row of each panel, plots of signal intensity vs. PDM in the respective channels and the corresponding intensity scatter plots are shown. PDM plots skewed to negative values demonstrate anti-correlation. In the third row, profiles of the signal intensities along the indicated lines also demonstrate underrepresentation of H3K4me3 and active RNA Pol II at damage sites.
(PDF)

S6 Fig Early formation of γH2AX foci is not affected by depletion of Jarid1A. 72 h after transfection with scr or Jarid1A A1+A3 siRNA, HeLa cells were irradiated in a small angle configuration with 55 MeV carbon ions and fixed after 5 min (A) and 2 min (B), or irradiated with 5 Gy X-rays and fixed after 15 min (C). Indirect immunofluorescence was performed to detect the foci formation of γH2AX. Numbers indicate microscopic exposure times, thus enabling direct comparison of signal intensities.
(TIF)

S7 Fig Background and residual 53BP1 and Rad51 foci are not affected by depletion of Jarid1A. Mean background frequency and number of residual 53BP1 and Rad51 foci (+/- SEM) were determined in at least 20 cells after 5 Gy X-irradiation. Cells were fixed before irradiation or at 24 h and 48 h after irradiation and indirect immunofluorescence was performed. Semi-automatic detection and characterization of foci was performed using the PlugIn FociPicker3D [28]. Differences between Jarid1 depletion and scr controls are not significant (p > 0.05).
(TIF)

S1 Table Comparison of relative band intensities corresponding to Jarid1A and Jarid1B proteins. Band intensities were normalized with respect to Tubulin loading control. Intensities in HeLa cells were set as 100%. All bands to be compared were on the same blot. Data are from 2 Western Blots.
(DOCX)

## References

[1] Baylin SB, Jones PA. A decade of exploring the cancer epigenome–biological and translational implications. Nat Rev Cancer. 2011;11: 726–734. 10.1038/nrc3130 21941284 PMC3307543

[2] Plass C, Pfister SM, Lindroth AM, Bogatyrova O, Claus R, Lichter P. Mutations in regulators of the epigenome and their connections to global chromatin patterns in cancer. Nat Rev Genet. 2013;14: 765–780. 10.1038/nrg3554 24105274

[3] Morgan MA, Shilatifard A. Chromatin signatures of cancer. Genes Dev. 2015;29: 238–249. 10.1101/gad.255182.114 25644600 PMC4318141

[4] You JS, Jones PA. Cancer genetics and epigenetics: two sides of the same coin? Cancer Cell. 2012;22: 9–20. 10.1016/j.ccr.2012.06.008 22789535 PMC3396881

[5] Højfeldt JW, Agger K, Helin K. Histone lysine demethylases as targets for anticancer therapy. Nat Rev Drug Discov. 2013;12: 917–930. 10.1038/nrd4154 24232376

[6] Mannironi C, Proietto M, Bufalieri F, Cundari E, Alagia A, Danovska S, et al An high-throughput in vivo screening system to select H3K4-specific histone demethylase inhibitors. PLoS One. 2014;9: e86002 10.1371/journal.pone.0086002 24489688 PMC3906020

[7] McGrath J, Trojer P. Targeting histone lysine methylation in cancer. Pharmacol Ther. 2015;150: 1–22. 10.1016/j.pharmthera.2015.01.002 25578037

[8] Chowdhury R, Yeoh KK, Tian YM, Hillringhaus L, Bagg EA, Rose NR, et al The oncometabolite 2-hydroxyglutarate inhibits histone lysine demethylases. EMBO Rep. 2011;12: 463–469. 10.1038/embor.2011.43 21460794 PMC3090014

[9] Xu W, Yang H, Liu Y, Yang Y, Wang P, Kim SH, et al Oncometabolite 2-hydroxyglutarate is a competitive inhibitor of α-ketoglutarate-dependent dioxygenases. Cancer Cell. 2011;19: 17–30. 10.1016/j.ccr.2010.12.014 21251613 PMC3229304

[10] Lu C, Ward PS, Kapoor GS, Rohle D, Turcan S, Abdel-Wahab O, et al IDH mutation impairs histone demethylation and results in a block to cell differentiation. Nature. 2012;483: 474–478. 10.1038/nature10860 22343901 PMC3478770

[11] Turcan S, Rohle D, Goenka A, Walsh LA, Fang F, Yilmaz E, et al IDH1 mutation is sufficient to establish the glioma hypermethylator phenotype. Nature. 2012;483: 479–483. 10.1038/nature10866 22343889 PMC3351699

[12] Kernytsky A, Wang F, Hansen E, Schalm S, Straley K, Gliser C, et al IDH2 mutation-induced histone and DNA hypermethylation is progressively reversed by small-molecule inhibition. Blood. 2015;125: 296–303. 10.1182/blood-2013-10-533604 25398940 PMC4295919

[13] Losman JA, Kaelin WG Jr. What a difference a hydroxyl makes: mutant IDH, (R)-2-hydroxyglutarate, and cancer. Genes Dev. 2013;27: 836–852. 10.1101/gad.217406.113 23630074 PMC3650222

[14] Li S, Chou AP, Chen W, Chen R, Deng Y, Phillips HS, et al Overexpression of isocitrate dehydrogenase mutant proteins renders glioma cells more sensitive to radiation. Neuro Oncol. 2013;15:57–68. 10.1093/neuonc/nos261 23115158 PMC3534418

[15] Wang XW, Labussière M, Valable S, Pérès EA, Guillamo JS, Bernaudin M, et al IDH1(R132H) mutation increases U87 glioma cell sensitivity to radiation therapy in hypoxia. Biomed Res Int. 2014;2014: 198697 10.1155/2014/198697 24895549 PMC4033346

[16] Young LC, McDonald DW, Hendzel MJ. Kdm4b histone demethylase is a DNA damage response protein and confers a survival advantage following γ-irradiation. J Biol Chem. 2013;288: 21376–21388. 10.1074/jbc.M113.491514 23744078 PMC3774405

[17] Li X, Liu L, Yang S, Song N, Zhou X, Gao J, et al Histone demethylase KDM5B is a key regulator of genome stability. Proc Natl Acad Sci U S A. 2014;111:7096–7101. 10.1073/pnas.1324036111 24778210 PMC4024858

[18] Khoury-Haddad H, Guttmann-Raviv N, Ipenberg I, Huggins D, Jeyasekharan AD, Ayoub N. PARP1-dependent recruitment of KDM4D histone demethylase to DNA damage sites promotes double-strand break repair. Proc Natl Acad Sci U S A. 2014;111: E728–737. 10.1073/pnas.1317585111 24550317 PMC3932863

[19] Khoury-Haddad H, Nadar-Ponniah PT, Awwad S, Ayoub N. The emerging role of lysine demethylases in DNA damage response: dissecting the recruitment mode of KDM4D/JMJD2D to DNA damage sites. Cell Cycle.;14: 950–958. 10.1080/15384101.2015.1014147 25714495 PMC4614868

[20] Hendriks IA, Treffers LW, Verlaan-de Vries M, Olsen JV, Vertegaal AC. SUMO-2 Orchestrates Chromatin Modifiers in Response to DNA Damage. Cell Rep. 2015; 10: 1778–1791. 10.1016/j.celrep.2015.02.033 PMC4514456 25772364

[21] Kuo YT, Liu YL, Adebayo BO, Shih PH, Lee WH, Wang LS, et al JARID1B Expression Plays a Critical Role in Chemoresistance and Stem Cell-Like Phenotype of Neuroblastoma Cells. PLoS One. 2015;10: e0125343 10.1371/journal.pone.0125343 25951238 PMC4423965

[22] Williams K, Christensen J, Rappsilber J, Nielsen AL, Johansen JV, Helin K. The histone lysine demethylase JMJD3/KDM6B is recruited to p53 bound promoters and enhancer elements in a p53 dependent manner. PLoS One. 2014;9: e96545 10.1371/journal.pone.0096545 24797517 PMC4010471

[23] Seiler DM, Rouquette J, Schmid VJ, Strickfaden H, Ottmann C, Drexler GA, et al Double-strand break-induced transcriptional silencing is associated with loss of tri-methylation at H3K4. Chromosome Res. 2011;19: 883–899. 10.1007/s10577-011-9244-1 21987186

[24] Grigaravicius P, Greulich KO, Monajembashi S. Laser microbeams and optical tweezers in ageing research. Chemphyschem. 2009;10:79–85. 10.1002/cphc.200800725 19090523

[25] Hauptner A, Dietzel S, Drexler GA, Reichart P, Krücken R, Cremer T, et al Microirradiation of cells with energetic heavy ions. Radiat Environ Biophys. 2004;42: 237–245. 14735370 10.1007/s00411-003-0222-7

[26] Hable V, Greubel C, Bergmaier A, Reichart P, Hauptner A, Krücken R, et al The live-cell irradiation and observation set-up at SNAKE. Nucl Instr and Meth in Phys Res B, 2009; 267: 2090–2097.

[27] Auer S, Hable V, Greubel C, Drexler GA, Schmid TE, Belka C, et al Survival of tumor cells after proton irradiation with ultra-high dose rates. Radiat Oncol. 2011;6: 139 10.1186/1748-717X-6-139 22008289 PMC3215966

[28] Du G, Drexler GA, Friedland W, Greubel C, Hable V, Krücken R, et al Spatial dynamics of DNA damage response protein foci along the ion trajectory of high-LET particles. Radiat Res. 2011;176: 706–715. 21797665 10.1667/rr2592.1

[29] Hable V, Drexler GA, Brüning T, Burgdorf C, Greubel C, Derer A, et al Recruitment kinetics of DNA repair proteins Mdc1 and Rad52 but not 53BP1 depend on damage complexity. PLoS One. 2012;7: e41943 10.1371/journal.pone.0041943 22860035 PMC3408406

[30] Girst S, Hable V, Drexler GA, Greubel C, Siebenwirth C, Haum M, et al Subdiffusion supports joining of correct ends during repair of DNA double-strand breaks. Sci Rep. 2013;3: 2511 10.1038/srep02511 23979012 PMC3753591

[31] Drexler GA, Siebenwirth C, Drexler SE, Girst S, Greubel C, Dollinger G, et al Live cell imaging at the Munich ion microbeam SNAKE—a status report. Radiat Oncol. 2015;10: 42 10.1186/s13014-015-0350-7 25880907 PMC4341815

[32] Zinner R, Teller K, Versteeg R, Cremer T, Cremer M. Biochemistry meets nuclear architecture: multicolor immuno-FISH for co-localization analysis of chromosome segments and differentially expressed gene loci with various histone methylations. Adv Enzyme Regul. 2007;47: 223–241. 17442381 10.1016/j.advenzreg.2007.01.005

[33] Greubel C, Hable V, Drexler GA, Hauptner A, Dietzel S, Strickfaden H, et al Competition effect in DNA damage response. Radiat Environ Biophys. 2008;47:423–429. 10.1007/s00411-008-0182-z 18648839

[34] R Core. R: A language and environment for statistical computing R Foundation for Statistical Computing, Vienna, Austria Team 2014 Available: http://www.R-project.org/.

[35] Braselmann H. CFAssay: Statistical analysis for the Colony Formation Assay. R package version 1.2.0. 2014. Available: http://www.bioconductor.org/packages/release/bioc/html/CFAssay.html.

[36] Faraway JJ. Extending the Linear Model with R: Generalized Linear, Mixed Effects and Nonparametric Regression Models. Chapman & Hall, Boca Raton, FL 2006

[37] Mansour WY, Schumacher S, Rosskopf R, Rhein T, Schmidt-Petersen F, Gatzemeier F, et al Hierarchy of nonhomologous end-joining, single-strand annealing and gene conversion at site-directed DNA double-strand breaks. Nucleic Acids Res. 2008;36: 4088–4098. 10.1093/nar/gkn347 18539610 PMC2475611

[38] Beshiri ML, Islam A, DeWaal DC, Richter WF, Love J, Lopez-Bigas N, et al Genome-wide analysis using ChIP to identify isoform-specific gene targets. J Vis Exp. 2010; 41 pii: 2101. 10.3791/2101 PMC3156076 20644511

[39] Islam AB, Richter WF, Lopez-Bigas N, Benevolenskaya EV. Selective targeting of histone methylation. Cell Cycle. 2011;10: 413–424. 21270517 10.4161/cc.10.3.14705 PMC3115016

[40] Chicas A, Kapoor A, Wang X, Aksoy O, Evertts AG, Zhang MQ, et al H3K4 demethylation by Jarid1a and Jarid1b contributes to retinoblastoma-mediated gene silencing during cellular senescence. Proc Natl Acad Sci U S A. 2012;109: 8971–8976. 10.1073/pnas.1119836109 22615382 PMC3384172

[41] Benevolenskaya EV, Murray HL, Branton P, Young RA, Kaelin WG Jr. Binding of pRB to the PHD protein RBP2 promotes cellular differentiation. Mol Cell. 2005;18: 623–635. 15949438 10.1016/j.molcel.2005.05.012

[42] Zeng J, Ge Z, Wang L, Li Q, Wang N, Björkholm M, et al The histone demethylase RBP2 Is overexpressed in gastric cancer and its inhibition triggers senescence of cancer cells. Gastroenterology. 2010;138:981–992. 10.1053/j.gastro.2009.10.004 19850045

[43] Teng YC, Lee CF, Li YS, Chen YR, Hsiao PW, Chan MY, et al Histone demethylase RBP2 promotes lung tumorigenesis and cancer metastasis. Cancer Res. 2013;73: 4711–4721. 10.1158/0008-5472.CAN-12-3165 23722541

[44] Liang X, Zeng J, Wang L, Fang M, Wang Q, Zhao M, et al Histone demethylase retinoblastoma binding protein 2 is overexpressed in hepatocellular carcinoma and negatively regulated by hsa-miR-212. PLoS One. 2013;8: e69784 10.1371/journal.pone.0069784 23922798 PMC3726779

[45] Li L, Wang L, Song P, Geng X, Liang X, Zhou M, et al Critical role of histone demethylase RBP2 in human gastric cancer angiogenesis. Mol Cancer. 2014;13: 81 10.1186/1476-4598-13-81 24716659 PMC4113143

[46] Hayakawa T, Ohtani Y, Hayakawa N, Shinmyozu K, Saito M, Ishikawa F, et al RBP2 is an MRG15 complex component and down-regulates intragenic histone H3 lysine 4 methylation. Genes Cells. 2007;12:811–826. 17573780 10.1111/j.1365-2443.2007.01089.x

[47] Nishibuchi G, Shibata Y, Hayakawa T, Hayakawa N, Ohtani Y, Sinmyozu K, et al Physical and functional interactions between the histone H3K4 demethylase KDM5A and the nucleosome remodeling and deacetylase (NuRD) complex. J Biol Chem. 2014;289: 28956–28970. 10.1074/jbc.M114.573725 25190814 PMC4200253

[48] Marcon E, Ni Z, Pu S, Turinsky AL, Trimble SS, Olsen JB, et al Human-chromatin-related protein interactions identify a demethylase complex required for chromosome segregation. Cell Rep. 2014;8: 297–310. 10.1016/j.celrep.2014.05.050 24981860

[49] Perry M, Chalkley R. The effect of histone hyperacetylation on the nuclease sensitivity and the solubility of chromatin. J Biol Chem. 1981;256: 3313–3318. 6259161

[50] Gilbert N, Thomson I, Boyle S, Allan J, Ramsahoye B, Bickmore WA. DNA methylation affects nuclear organization, histone modifications, and linker histone binding but not chromatin compaction. J Cell Biol. 2007;177: 401–411. 17485486 10.1083/jcb.200607133 PMC2064831

[51] Miller KM, Tjeertes JV, Coates J, Legube G, Polo SE, Britton S, et al Human HDAC1 and HDAC2 function in the DNA-damage response to promote DNA nonhomologous end-joining. Nat Struct Mol Biol. 2010;17: 1144–1151. 10.1038/nsmb.1899 20802485 PMC3018776

[52] Groselj B, Sharma NL, Hamdy FC, Kerr M, Kiltie AE. Histone deacetylase inhibitors as radiosensitisers: effects on DNA damage signalling and repair. Br J Cancer. 2013;108: 748–54. 10.1038/bjc.2013.21 23361058 PMC3590661

[53] Karagiannis TC, Harikrishnan KN, El-Osta A. Disparity of histone deacetylase inhibition on repair of radiation-induced DNA damage on euchromatin and constitutive heterochromatin compartments. Oncogene. 2007;26: 3963–3971. 17213813 10.1038/sj.onc.1210174

[54] Hsiao KY, Mizzen CA. Histone H4 deacetylation facilitates 53BP1 DNA damage signaling and double-strand break repair. J Mol Cell Biol. 2013;5: 157–165. 10.1093/jmcb/mjs066 23329852

[55] Tang J, Cho NW, Cui G, Manion EM, Shanbhag NM, Botuyan MV, et al Acetylation limits 53BP1 association with damaged chromatin to promote homologous recombination. Nat Struct Mol Biol. 2013;20: 317–325 10.1038/nsmb.2499 23377543 PMC3594358

[56] Larsen DH, Poinsignon C, Gudjonsson T, Dinant C, Payne MR, Hari FJ, et al The chromatin-remodeling factor CHD4 coordinates signaling and repair after DNA damage. J Cell Biol. 2010;190: 731–740. 10.1083/jcb.200912135 20805324 PMC2935572

[57] Jakob B, Scholz M, Taucher-Scholz G. Biological imaging of heavy charged-particle tracks. Radiat Res. 2003;159:676–684. 12710880 10.1667/0033-7587(2003)159[0676:biohct]2.0.co;2

[58] Daley JM, Sung P. 53BP1, BRCA1, and the choice between recombination and end joining at DNA double-strand breaks. Mol Cell Biol. 2014;34: 1380–1388. 10.1128/MCB.01639-13 24469398 PMC3993578

[59] Löbrich M, Shibata A, Beucher A, Fisher A, Ensminger M, Goodarzi AA, et al gammaH2AX foci analysis for monitoring DNA double-strand break repair: strengths, limitations and optimization. Cell Cycle. 2010;9: 662–669. 20139725 10.4161/cc.9.4.10764

[60] Mariotti LG, Pirovano G, Savage KI, Ghita M, Ottolenghi A, Prise KM, et al Use of the γ-H2AX assay to investigate DNA repair dynamics following multiple radiation exposures. PLoS One. 2013;8: e79541 10.1371/journal.pone.0079541 24312182 PMC3843657

[61] Mansour WY, Rhein T, Dahm-Daphi J. The alternative end-joining pathway for repair of DNA double-strand breaks requires PARP1 but is not dependent upon microhomologies. Nucleic Acids Res. 2010;38: 6065–6077. 10.1093/nar/gkq387 20483915 PMC2952854

[62] Mansour WY, Borgmann K, Petersen C, Dikomey E, Dahm-Daphi J. The absence of Ku but not defects in classical non-homologous end-joining is required to trigger PARP1-dependent end-joining. DNA Repair (Amst). 2013;12: 1134–1142. 24210699 10.1016/j.dnarep.2013.10.005

[63] Hou J, Wu J, Dombkowski A, Zhang K, Holowatyj A, Boerner JL, et al Genomic amplification and a role in drug-resistance for the KDM5A histone demethylase in breast cancer. Am J Transl Res. 2012;4: 247–256. 22937203 PMC3426386

[64] Klein BJ, Piao L, Xi Y, Rincon-Arano H, Rothbart SB, Peng D, et al The histone-H3K4-specific demethylase KDM5B binds to its substrate and product through distinct PHD fingers. Cell Rep. 2014;6: 325–335. 10.1016/j.celrep.2013.12.021 24412361 PMC3918441

[65] Xie L, Pelz C, Wang W, Bashar A, Varlamova O, Shadle S, et al KDM5B regulates embryonic stem cell self-renewal and represses cryptic intragenic transcription. EMBO J. 2011;30: 1473–1484. 10.1038/emboj.2011.91 21448134 PMC3102288

[66] Outchkourov NS, Muiño JM, Kaufmann K, van Ijcken WF, Groot Koerkamp MJ, van Leenen D, et al Balancing of histone H3K4 methylation states by the Kdm5c/SMCX histone demethylase modulates promoter and enhancer function. Cell Rep. 2013;3:1071–1079. 10.1016/j.celrep.2013.02.030 23545502

[67] Sharma SV, Lee DY, Li B, Quinlan MP, Takahashi F, Maheswaran S, et al A chromatin-mediated reversible drug-tolerant state in cancer cell subpopulations. Cell. 2010;141: 69–80. 10.1016/j.cell.2010.02.027 20371346 PMC2851638

[68] Klose RJ, Yan Q, Tothova Z, Yamane K, Erdjument-Bromage H, Tempst P, et al The retinoblastoma binding protein RBP2 is an H3K4 demethylase. Cell. 2007;128: 889–900. 17320163 10.1016/j.cell.2007.02.013

[69] Pasini D, Hansen KH, Christensen J, Agger K, Cloos PA, Helin K. Coordinated regulation of transcriptional repression by the RBP2 H3K4 demethylase and Polycomb-Repressive Complex 2. Genes Dev. 2008;22: 1345–1355. 10.1101/gad.470008 18483221 PMC2377189

[70] Kralj M, Husnjak K, Körbler T, Pavelić J. Endogenous p21WAF1/CIP1 status predicts the response of human tumor cells to wild-type p53 and p21WAF1/CIP1 overexpression. Cancer Gene Ther. 2003;10: 457–467. 12768191 10.1038/sj.cgt.7700588

[71] Chang LJ, Eastman A. Differential regulation of p21 (waf1) protein half-life by DNA damage and Nutlin-3 in p53 wild-type tumors and its therapeutic implications. Cancer Biol Ther. 2012;13: 1047–1057. 10.4161/cbt.21047 22825333 PMC3461812

[72] Latham JA, Dent SY. Cross-regulation of histone modifications. Nat Struct Mol Biol. 2007;14:1017–1024. 17984964 10.1038/nsmb1307

[73] Vasilatos SN, Katz TA, Oesterreich S, Wan Y, Davidson NE, Huang Y. Crosstalk between lysine-specific demethylase 1 (LSD1) and histone deacetylases mediates antineoplastic efficacy of HDAC inhibitors in human breast cancer cells. Carcinogenesis. 2013;34: 1196–1207. 10.1093/carcin/bgt033 23354309 PMC3670252

[74] Yin F, Lan R, Zhang X, Zhu L, Chen F, Xu Z, et al LSD1 regulates pluripotency of embryonic stem/carcinoma cells through histone deacetylase 1-mediated deacetylation of histone H4 at lysine 16. Mol Cell Biol. 2014;34: 158–179. 10.1128/MCB.00631-13 24190971 PMC3911295

[75] Huang PH, Chen CH, Chou CC, Sargeant AM, Kulp SK, Teng CM, et al Histone deacetylase inhibitors stimulate histone H3 lysine 4 methylation in part via transcriptional repression of histone H3 lysine 4 demethylases. Mol Pharmacol. 2011;79: 197–206. 10.1124/mol.110.067702 20959362 PMC3014276

[76] Huang PH, Plass C, Chen CS. Effects of Histone Deacetylase Inhibitors on Modulating H3K4 Methylation Marks—A Novel Cross-Talk Mechanism between Histone-Modifying Enzymes. Mol Cell Pharmacol. 2011;3: 39–43. 22468166 PMC3315589

[77] Ganai SA, Kalladi SM, Mahadevan V. HDAC inhibition through valproic acid modulates the methylation profiles in human embryonic kidney cells. J Biomol Struct Dyn. 2015;33: 1185–97. 10.1080/07391102.2014.938247 25012937

[78] Wu S, Ge Y, Huang L, Liu H, Xue Y, Zhao Y. BRG1, the ATPase subunit of SWI/SNF chromatin remodeling complex, interacts with HDAC2 to modulate telomerase expression in human cancer cells. Cell Cycle. 2014;13: 2869–2878. 10.4161/15384101.2014.946834 25486475 PMC4612678

[79] Huttlin EL, Ting L, Bruckner RJ, Gebreab F, Gygi MP, Szpyt J, et al The BioPlex Network: A Systematic Exploration of the Human Interactome. Cell. 2015;162: 425–440. 10.1016/j.cell.2015.06.043 26186194 PMC4617211

[80] Bhaskara S, Jacques V, Rusche JR, Olson EN, Cairns BR, Chandrasekharan MB. Histone deacetylases 1 and 2 maintain S-phase chromatin and DNA replication fork progression. Epigenetics Chromatin. 2013;6: 27 10.1186/1756-8935-6-27 23947532 PMC3765969

[81] Goodarzi AA, Kurka T, Jeggo PA. KAP-1 phosphorylation regulates CHD3 nucleosome remodeling during the DNA double-strand break response. Nat Struct Mol Biol. 2011;18:831–839. 10.1038/nsmb.2077 21642969

[82] Dorigo B, Schalch T, Bystricky K, Richmond TJ. Chromatin fiber folding: requirement for the histone H4 N-terminal tail. J Mol Biol. 2003;327: 85–96. 12614610 10.1016/s0022-2836(03)00025-1

[83] Shogren-Knaak M, Ishii H, Sun JM, Pazin MJ, Davie JR, Peterson CL. Histone H4-K16 acetylation controls chromatin structure and protein interactions. Science. 2006;311: 844–847. 16469925 10.1126/science.1124000

[84] Taylor GC, Eskeland R, Hekimoglu-Balkan B, Pradeepa MM, Bickmore WA. H4K16 acetylation marks active genes and enhancers of embryonic stem cells, but does not alter chromatin compaction. Genome Res. 2013;23: 2053–2065. 10.1101/gr.155028.113 23990607 PMC3847775

[85] Cowell IG, Sunter NJ, Singh PB, Austin CA, Durkacz BW, Tilby MJ. gammaH2AX foci form preferentially in euchromatin after ionising-radiation. PLoS One. 2007;2: e1057 17957241 10.1371/journal.pone.0001057 PMC2020439

[86] Kim JA, Kruhlak M, Dotiwala F, Nussenzweig A, Haber JE. Heterochromatin is refractory to gamma-H2AX modification in yeast and mammals. J Cell Biol. 2007;178: 209–218. 17635934 10.1083/jcb.200612031 PMC2064441

[87] Vasireddy RS, Tang MM, Mah LJ, Georgiadis GT, El-Osta A, Karagiannis TC. Evaluation of the spatial distribution of gammaH2AX following ionizing radiation. J Vis Exp. 2010; 42 pii: 2203. 10.3791/2203 PMC3156025 20736911

[88] Takata H, Hanafusa T, Mori T, Shimura M, Iida Y, Ishikawa K, et al Chromatin compaction protects genomic DNA from radiation damage. PLoS One. 2013;8: e75622 10.1371/journal.pone.0075622 24130727 PMC3794047

[89] Kawano T, Akiyama M, Agawa-Ohta M, Mikami-Terao Y, Iwase S, Yanagisawa T, et al Histone deacetylase inhibitors valproic acid and depsipeptide sensitize retinoblastoma cells to radiotherapy by increasing H2AX phosphorylation and p53 acetylation-phosphorylation. Int J Oncol. 2010;37: 787–795. 20811699 10.3892/ijo_00000728

[90] Blattmann C, Oertel S, Thiemann M, Weber KJ, Schmezer P, Zelezny O, et al Suberoylanilide hydroxamic acid affects γH2AX expression in osteosarcoma, atypical teratoid rhabdoid tumor and normal tissue cell lines after irradiation. Strahlenther Onkol. 2012;188: 168–176. 10.1007/s00066-011-0028-5 22249335

[91] Makita N, Ninomiya I, Tsukada T, Okamoto K, Harada S, Nakanuma S, et al Inhibitory effects of valproic acid in DNA double-strand break repair after irradiation in esophageal squamous carcinoma cells. Oncol Rep. 2015;34: 1185–1192. 10.3892/or.2015.4089 26135807

[92] Fukuda T, Wu W, Okada M, Maeda I, Kojima Y, Hayami R, et al Class I histone deacetylase inhibitors inhibit the retention of BRCA1 and 53BP1 at the site of DNA damage. Cancer Sci. 2015;106:1050–1056. 10.1111/cas.12717 26053117 PMC4556395

[93] Neumayer G, Nguyen MD. TPX2 impacts acetylation of histone H4 at lysine 16: implications for DNA damage response. PLoS One. 2014;9: e110994 10.1371/journal.pone.0110994 25365214 PMC4217740

[94] Reindl J, Drexler GA, Girst S, Greubel C, Siebenwirth C, Drexler SE, et al Nanoscopic exclusion between Rad51 and 53BP1 after ion irradiation in human HeLa cells. Phys Biol. 2015;12: 066005 10.1088/1478-3975/12/6/066005 26595336

[95] Nagy Z, Soutoglou E. DNA repair: easy to visualize, difficult to elucidate. Trends Cell Biol. 2009;19: 617–629. 10.1016/j.tcb.2009.08.010 19819145

[96] Splinter J, Jakob B, Lang M, Yano K, Engelhardt J, Hell SW, et al Biological dose estimation of UVA laser microirradiation utilizing charged particle-induced protein foci. Mutagenesis. 2010;25: 289–297. 10.1093/mutage/geq005 20167590 PMC2902920

[97] Drexler GA, Ruiz-Gómez MJ. Microirradiation techniques in radiobiological research. J Biosci. 2015;40: 629–643. 26333407 10.1007/s12038-015-9535-3

[98] Khurana S, Kruhlak MJ, Kim J, Tran AD, Liu J, Nyswaner K, et al A macrohistone variant links dynamic chromatin compaction to BRCA1-dependent genome maintenance. Cell Rep. 2014;8: 1049–1062. 10.1016/j.celrep.2014.07.024 25131201 PMC4154351

[99] Murr R, Loizou JI, Yang YG, Cuenin C, Li H, Wang ZQ, Herceg Z. Histone acetylation by Trrap-Tip60 modulates loading of repair proteins and repair of DNA double-strand breaks. Nat Cell Biol. 2006;8: 91–99. 16341205 10.1038/ncb1343

