# Supplementary figures and images for: Depletion of Histone Demethylase Jarid1A Resulting in Histone Hyperacetylation and Radiation Sensitivity Does Not Affect DNA Double-Strand Break Repair
Source: PLoS One. 2016 Jun 2;11(6):e0156599. doi: 10.1371/journal.pone.0156599 (PMC4890786; doi:10.1371/journal.pone.0156599)

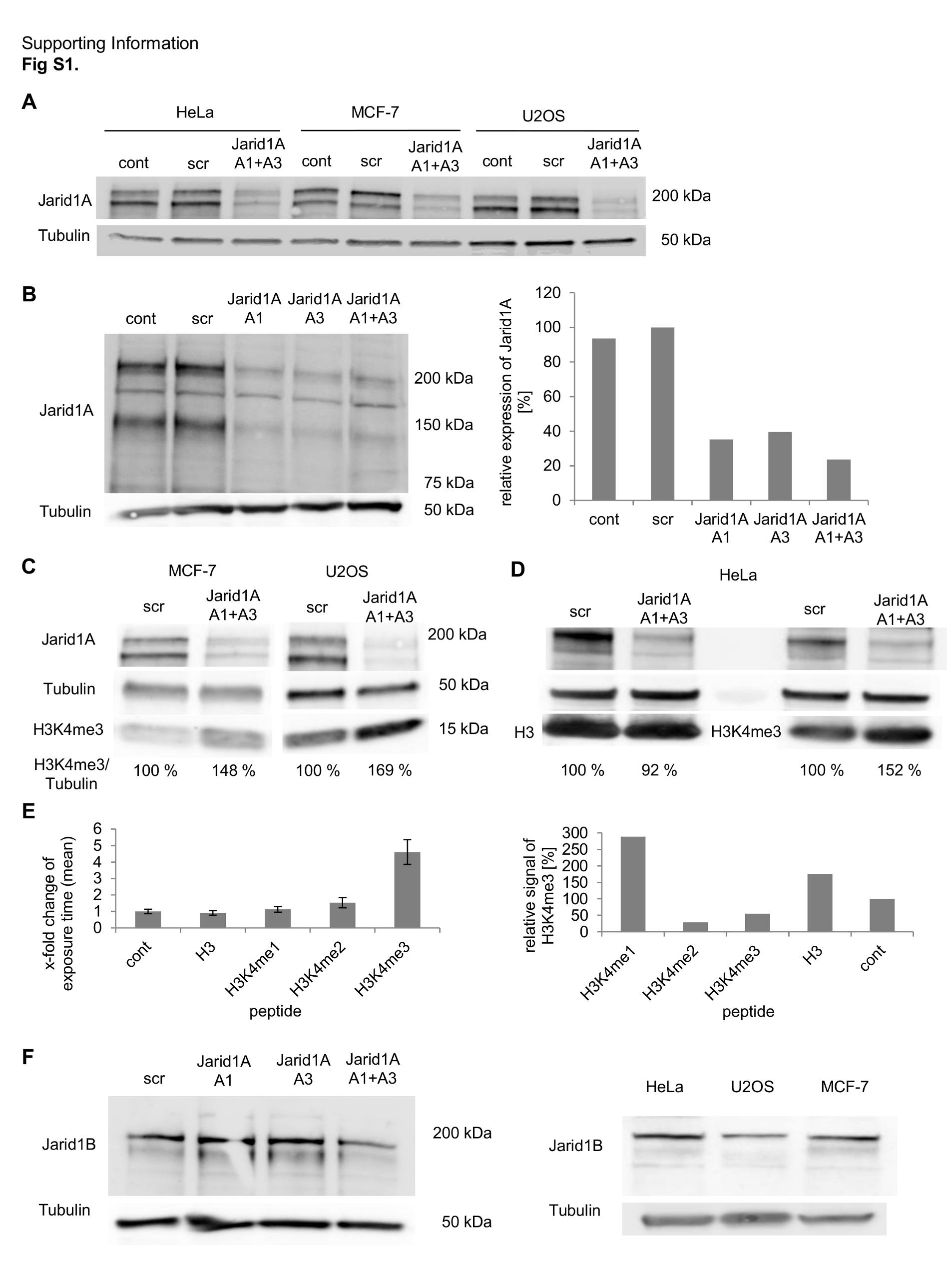

Supplement: S1 Fig — (A) Decreased levels of Jarid1A in whole cell protein extracts of HeLa, MCF-7 and U2OS cells after siRNA transfection. Western blot images show levels of Jarid1A in untransfected cells (cont), in cells transfected with scrambled siRNA (scr) and in cells transfected with Jarid1A A1+A3 siRNA. (B) Highest knock-down efficiency is accomplished with a combination of the siRNAs Jarid1A A1 and A3. An unspecific band at about 170 kD seen in certain gel settings is not affected by siRNA transfection. The graph depicts results of quantitative evaluation of the blot shown on the left side. (C) Increase of H3K4me3 in whole cell protein extracts of MCF-7 cells and U2OS cells after knock-down of Jarid1A. Western blot images show levels of Jarid1A and H3K4me3 in cells transfected with scr siRNA and Jarid1A A1+A3 siRNA 72 h after transfection. Numbers give levels of H3K4me3 normalized to scr sample after quantitative analysis. (D) Increase of H3K4me3 after Jarid1A depletion is not due to alterations in level of histone H3. Parallel samples of protein extracts of cells transfected with scr siRNA and Jarid1A A1+A3 siRNA were loaded. The low molecular weight region of the Western Blot was cut into 2 halves to visualize H3 and H3K4me3. Numbers give levels of H3K4me3 normalized to scr sample after quantitative analysis. (E) Specificity of the H3K4me3 antibody was established by peptide competition assays via immunofluorescence and Western Blot. The graph at the left shows the mean x-fold change of exposure time (+/-SD) from 5 randomly chosen positions in the immunofluorescence samples after incubation of the antibody with different peptides. Efficient blocking of the antibody is only seen with H3K4me3 peptides. For the Western Blot, the relative signals of H3K4me3 after incubation of the antibody with the different peptides were calculated and are represented in the right graph. The antibody is efficiently blocked by peptides H3K4me2 and H3K4me3. (F) Left panel: Level of Jarid1B protein [file pone.0156599.s001.tif]

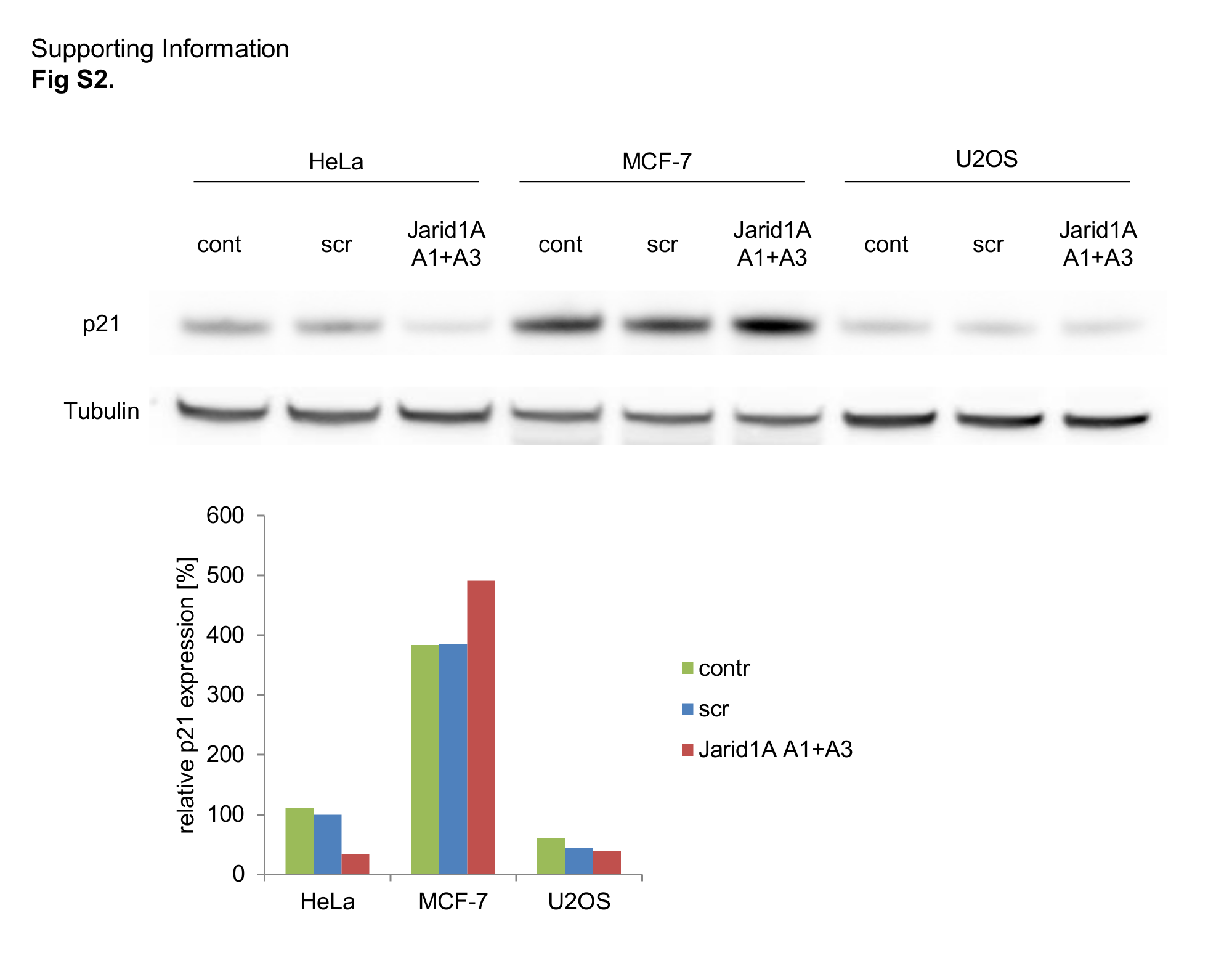

Supplement: S2 Fig — Expression of p21 after depletion of Jarid1A in HeLa, MCF-7 and U2OS cells, 72 h after transfection with scr or Jarid1A siRNA. A representative sample and its quantitative evaluation are shown. Cont = untransfected control. (TIF) [file pone.0156599.s002.tif]

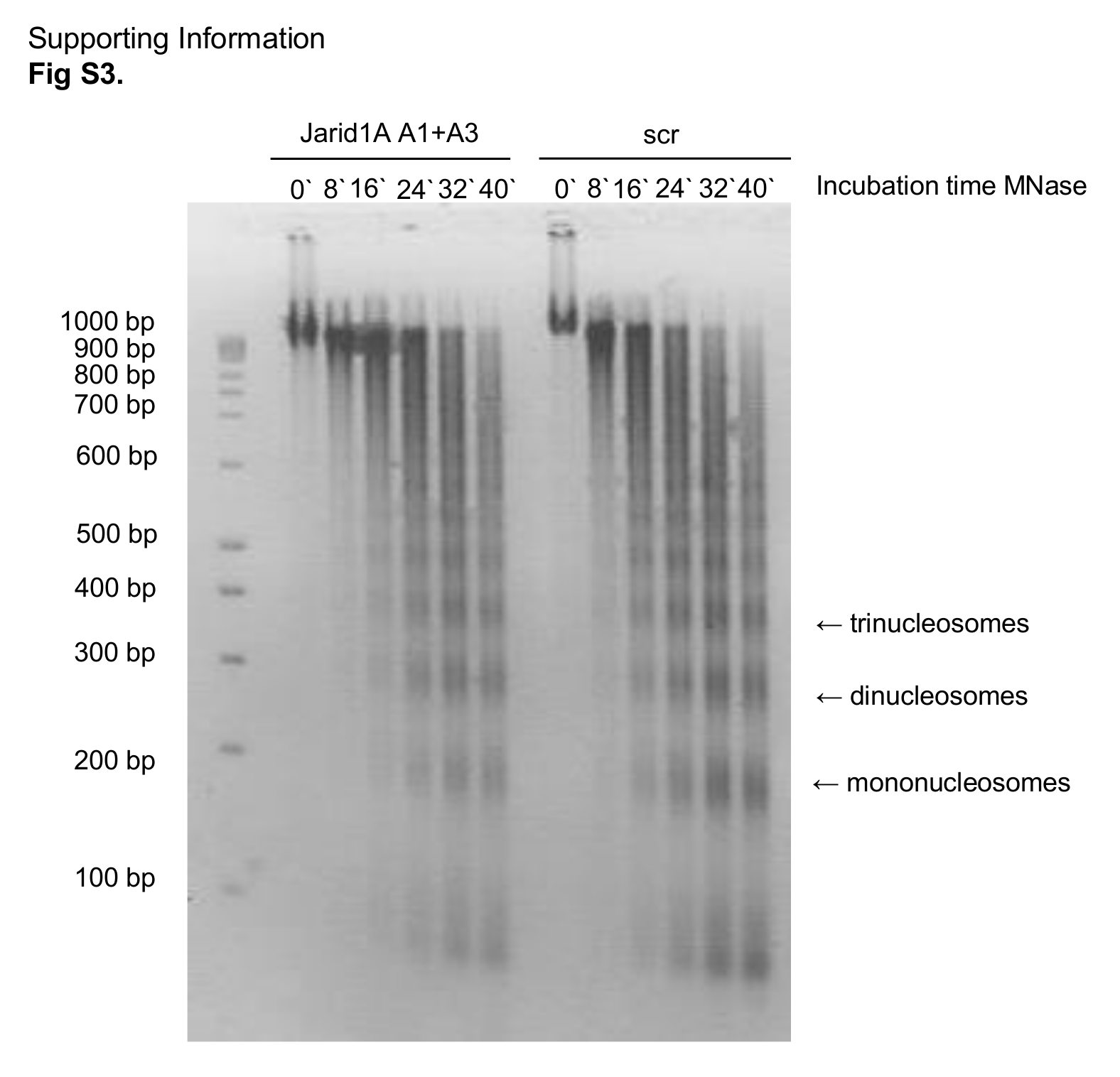

Supplement: S3 Fig — Analysis of chromatin accessibility by MNase digestion of isolated nuclei. After different incubation periods with 0.5 u MNase, comparable amounts of partially digested DNA were loaded onto an agarose gel. The emerging ladder of mono- and oligonucleosomes is comparable in both samples indicating regular nucleosome distribution in bulk chromatin after Jarid1A depletion. (TIF) [file pone.0156599.s003.tif]

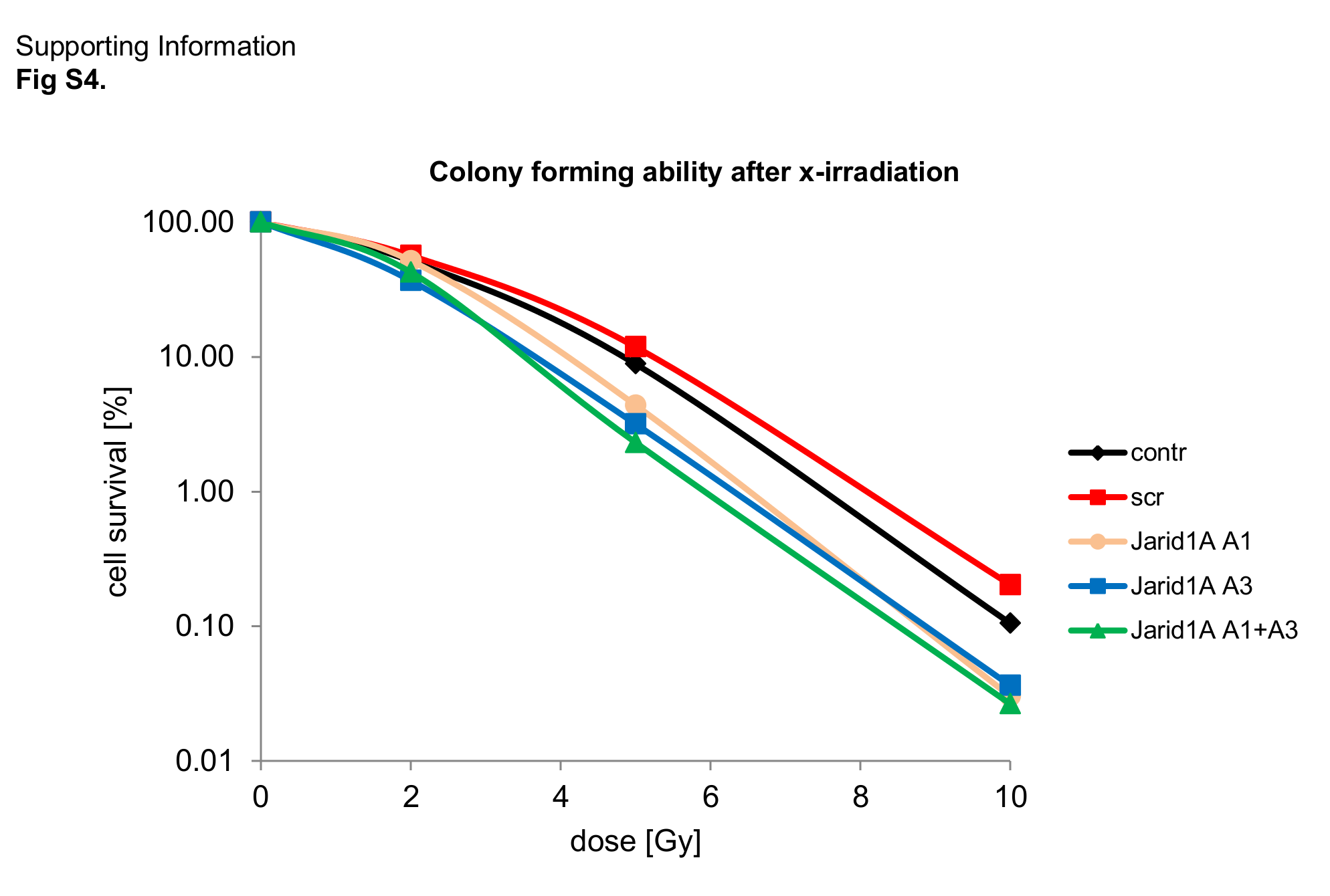

Supplement: S4 Fig — Colony formation experiment with Hela cells transfected with JaridA1 siRNAs A1 or A3 or a combination thereof, as well as untransfected controls and cells transfected with scr siRNA. Cells were irradiated 72 h after siRNA transfection with 0 Gy, 2 Gy, 5 Gy or 10 Gy X-rays. Cells were incubated for 10 days before fixation and methylene blue staining of colonies. Data show that the different Jarid1A siRNAs lead to comparable sensitization as compared to controls. (TIF) [file pone.0156599.s004.tif]

**scr**

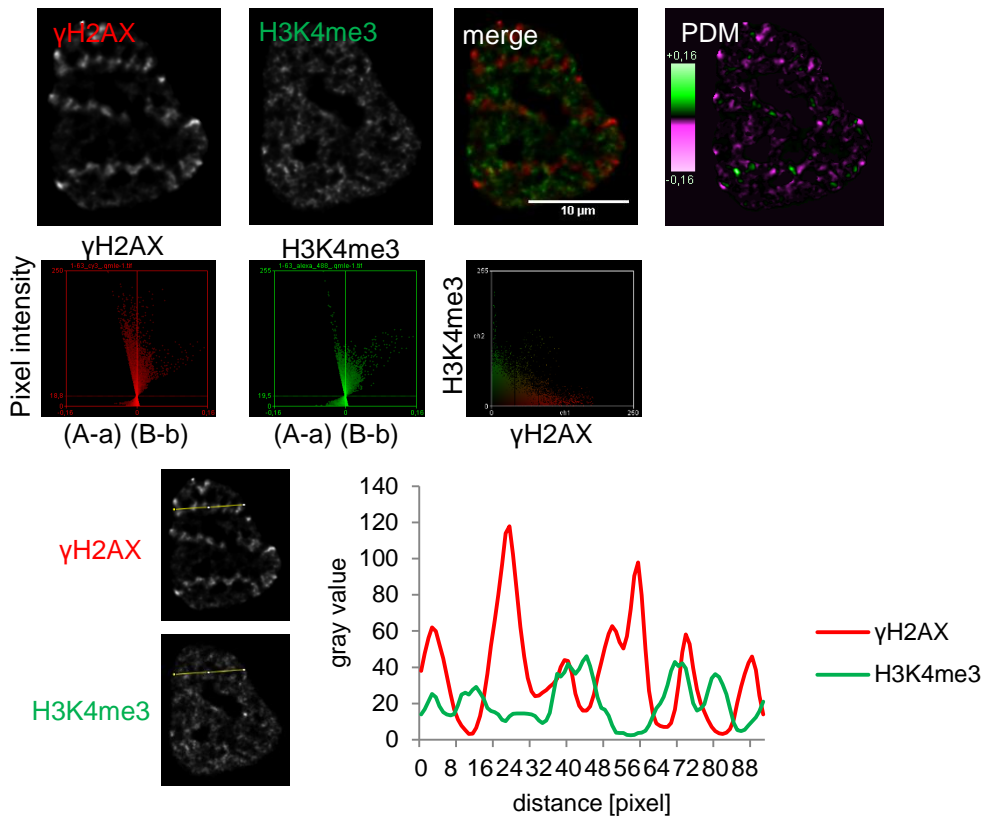

**Jarid1A A1+A3**

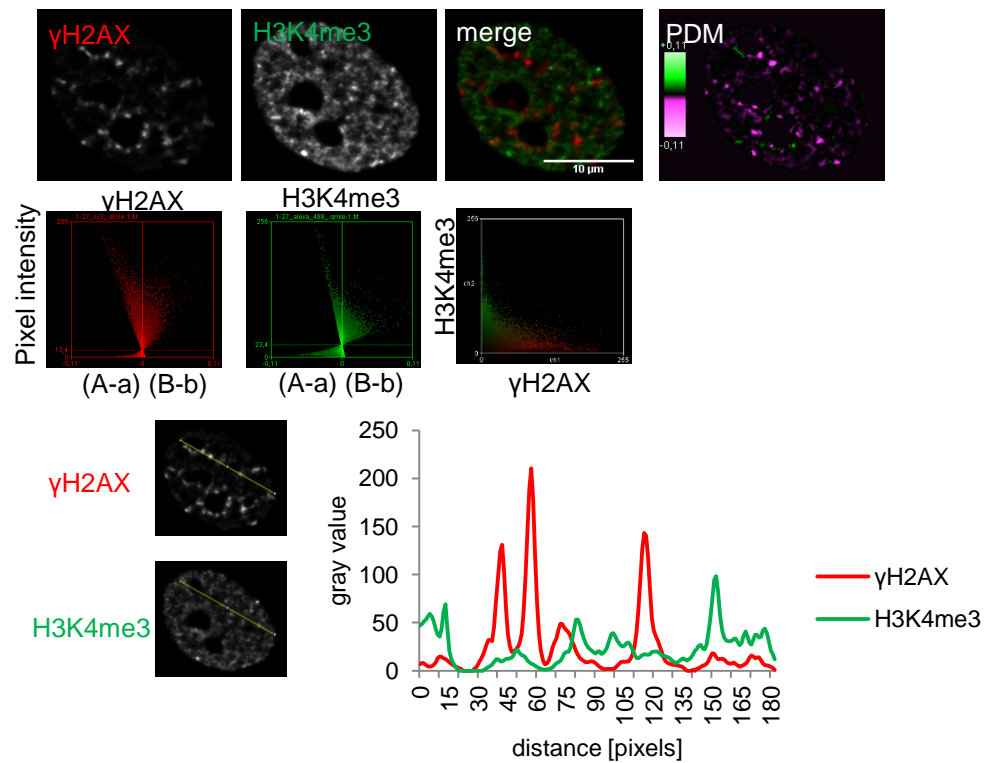

scr

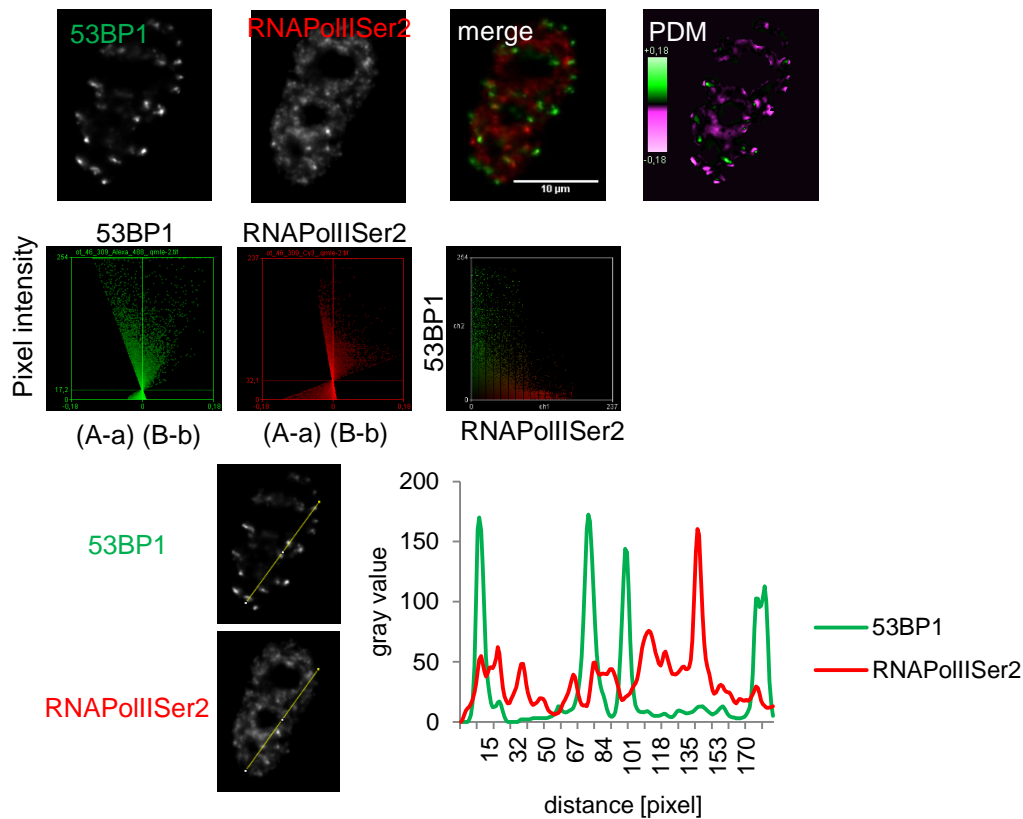

Jarid1A A1+A3

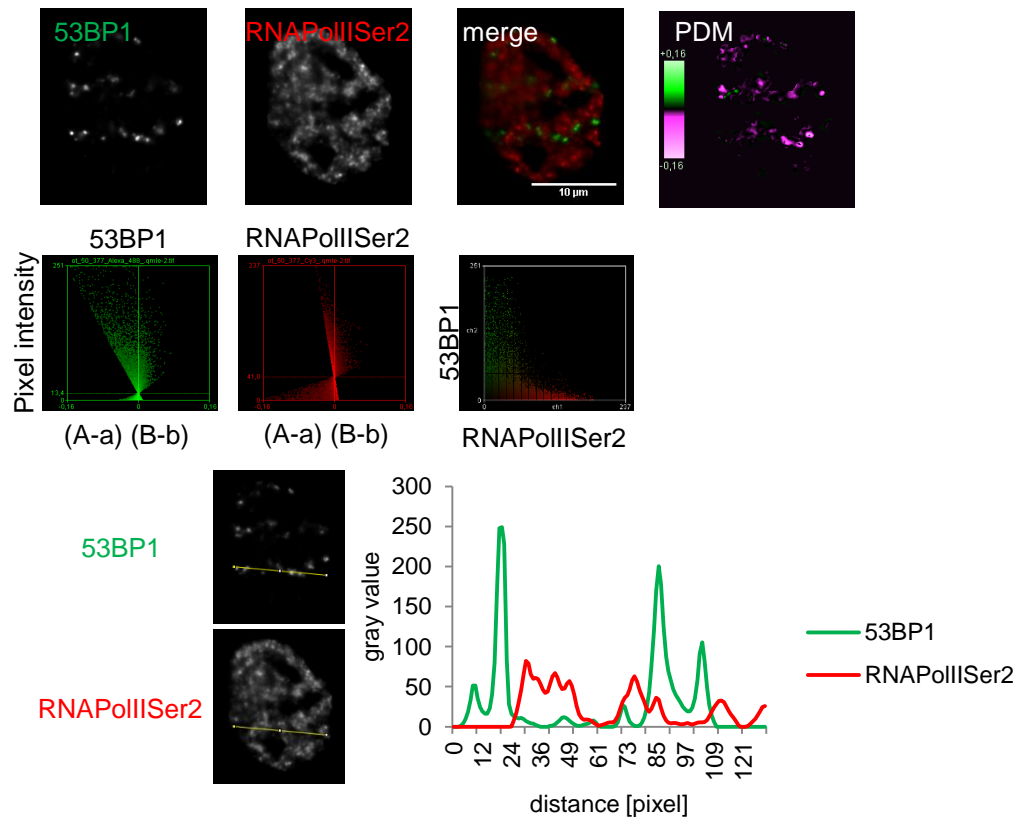

Supplement: S5 Fig — HeLa cell transfected with scr or Jarid1A A1+A3 siRNAs were subject to ion microirradiation with single carbon ions applied in line patterns (lateral distance between single ion hits 1 μm, distance between “lines” 5 μm). Cells were incubated for 1 h before fixation and indirect immunofluorescence detection of γH2AX and H3K4me3 (A) or elongation-proficient RNA Pol II Ser2-p (B). Correlation analysis was done as described [23]. In all panels the top rows show single slices of 3D microscopic images (red channel, green channel and merge). In addition, to determine positive or negative correlation between signal intensities in both channels for each pixel, the product of the mean (PDM) map is shown. In the PDM maps, negative correlation at positions of γH2AX foci is visualized by pink signals; positive correlation is shown by green signals, whereas black indicates random distribution of both signals. In the second row of each panel, plots of signal intensity vs. PDM in the respective channels and the corresponding intensity scatter plots are shown. PDM plots skewed to negative values demonstrate anti-correlation. In the third row, profiles of the signal intensities along the indicated lines also demonstrate underrepresentation of H3K4me3 and active RNA Pol II at damage sites. (PDF) [file pone.0156599.s005.pdf]

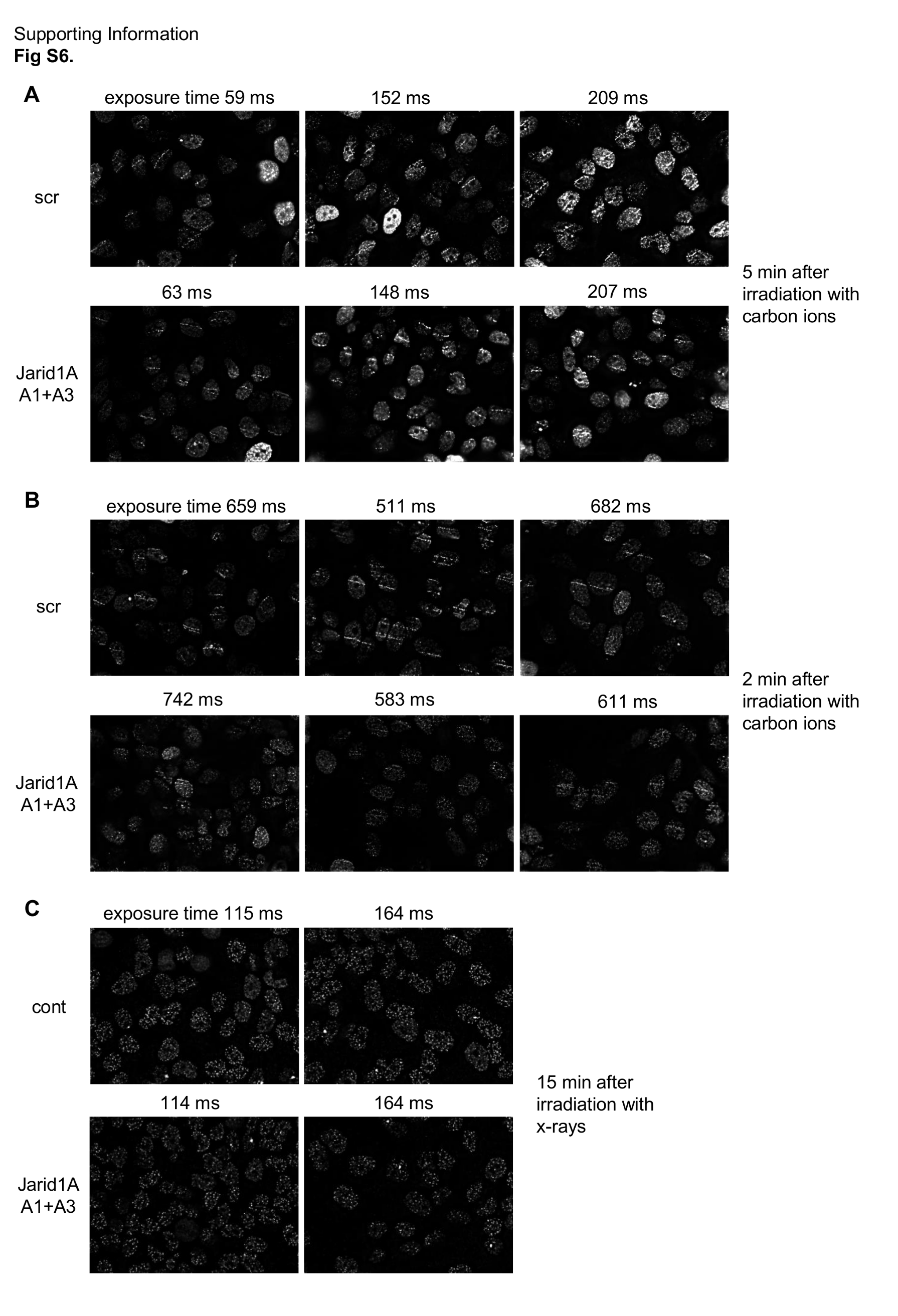

Supplement: S6 Fig — 72 h after transfection with scr or Jarid1A A1+A3 siRNA, HeLa cells were irradiated in a small angle configuration with 55 MeV carbon ions and fixed after 5 min (A) and 2 min (B), or irradiated with 5 Gy X-rays and fixed after 15 min (C). Indirect immunofluorescence was performed to detect the foci formation of γH2AX. Numbers indicate microscopic exposure times, thus enabling direct comparison of signal intensities. (TIF) [file pone.0156599.s006.tif]

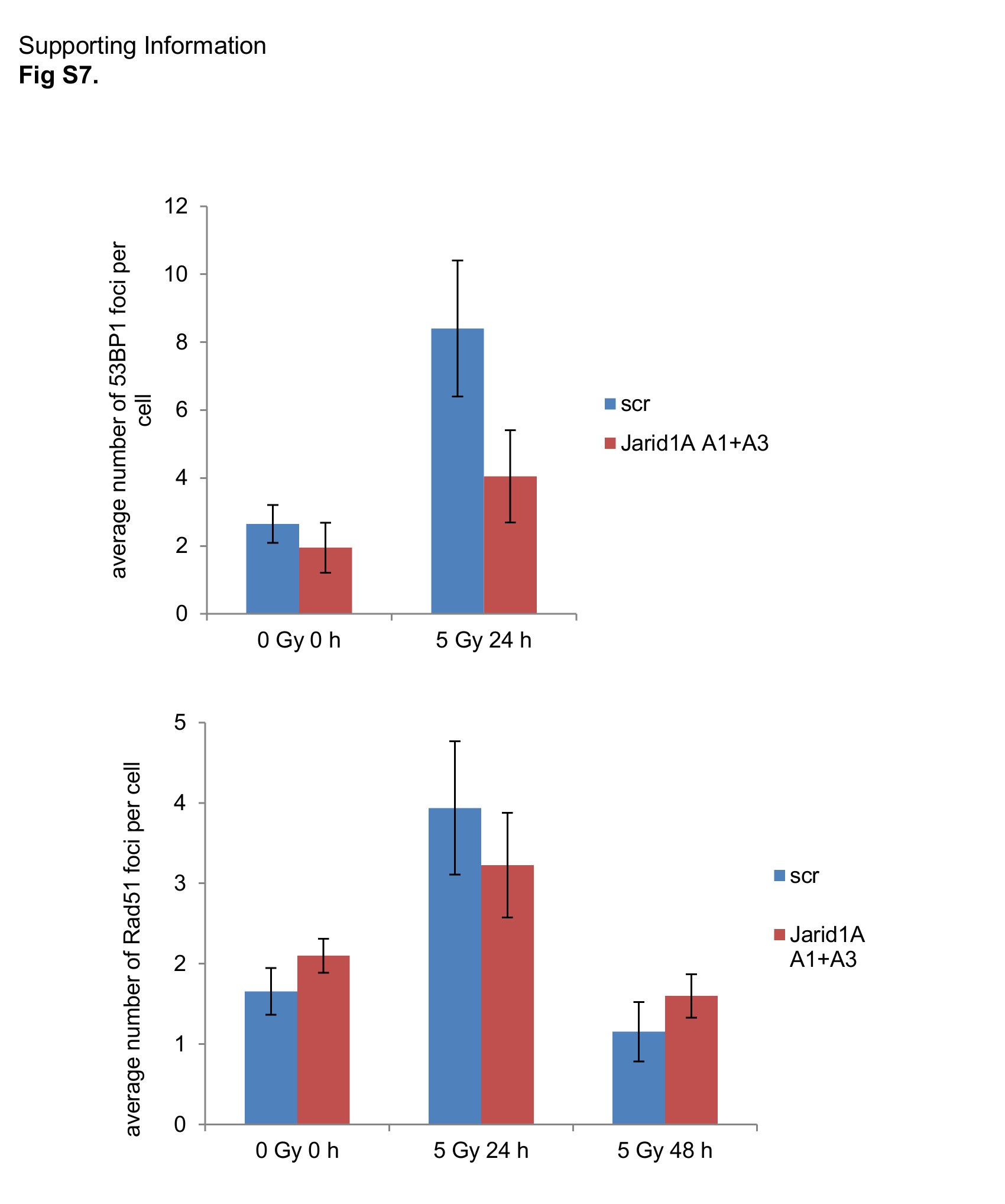

Supplement: S7 Fig — Mean background frequency and number of residual 53BP1 and Rad51 foci (+/- SEM) were determined in at least 20 cells after 5 Gy X-irradiation. Cells were fixed before irradiation or at 24 h and 48 h after irradiation and indirect immunofluorescence was performed. Semi-automatic detection and characterization of foci was performed using the PlugIn FociPicker3D [28]. Differences between Jarid1 depletion and scr controls are not significant (p > 0.05). (TIF) [file pone.0156599.s007.tif]
