# Supplementary material for: Depletion of Histone Demethylase Jarid1A Resulting in Histone Hyperacetylation and Radiation Sensitivity Does Not Affect DNA Double-Strand Break Repair
Source: PLoS One. 2016 Jun 2;11(6):e0156599. doi: 10.1371/journal.pone.0156599 (PMC4890786; doi:10.1371/journal.pone.0156599)
Supplement: S1 Table — Band intensities were normalized with respect to Tubulin loading control. Intensities in HeLa cells were set as 100%. All bands to be compared were on the same blot. Data are from 2 Western Blots. (DOCX) [file pone.0156599.s008.docx]

Supporting information

**S1 Table**

|  | HeLa | MCF-7 | U2OS |
| --- | --- | --- | --- |
| relative band intensity Jarid1A | 1.00 ± 0 | 1.00 ± 0.42 | 0.43 ± 0.23 |
| relative band intensity Jarid1B | 1.00 ± 0 | 0.99 ± 0.20 | 0.48 ± 0.02 |
| ratio | 1.00 | 1.01 | 0.89 |

Comparison of relative band intensities corresponding to Jarid1A and Jarid1B proteins after normalization with Tubulin loading control. Intensities in HeLa cells were set as 100%. All bands to be compared were on the same blot. Data are from 2 Western Blots.
